# Changes in the working conditions and learning environment of medical residents after the enactment of the Medical Resident Act in Korea in 2015: a national 4-year longitudinal study

**DOI:** 10.3352/jeehp.2021.18.7

**Published:** 2021-04-20

**Authors:** Sangho Sohn, Yeonjoo Seo, Yunsik Jeong, Seungwoo Lee, Jeesun Lee, Kyung Ju Lee

**Affiliations:** 1 Department of Preventive Medicine, Korea University College of Medicine, Seoul, Korea; 2 Department of Internal Medicine, College of Medicine, The Catholic University of Korea, Seoul, Korea; 3 Damyang-gun Public Health Care Center, Damyang, Korea; 4 Department of Psychiatry, Dankook University College of Medicine, Cheonan, Korea; 5 Korean Intern Resident Association, Seoul, Korea; 6 Department of Obstetrics and Gynecology, Korea University College of Medicine, Seoul, Korea; 7 Institute for Occupational and Environmental Health, Korea University, Seoul, Korea; Hallym University, Korea

**Keywords:** Internship and residency, Perception, Personal satisfaction, Republic of Korea, Training support

## Abstract

**Purpose:**

In 2015, the South Korean government legislated the Act for the Improvement of Training Conditions and Status of Medical Residents (Medical Resident Act). This study investigated changes in the working and learning environment pre- and post-implementation of the Medical Resident Act in 2017, as well as changes in training conditions by year post-implementation.

**Methods:**

An annual cross-sectional voluntary survey was conducted by the Korean Intern Resident Association (KIRA) between 2016 and 2019. The learning and working environment, including extended shift length, rest time, learning goals, and job satisfaction, were compared by institution type, training year, and specialty.

**Results:**

Of the 55,727 enrollees in the KIRA, 15,029 trainees took the survey, and the number of survey participants increased year by year (from 2,984 in 2016 to 4,700 in 2019). Overall working hours tended to decrease; however, interns worked the most (114 hours in 2016, 88 hours in 2019; P<0.001). Having 10 hours or more of break time has gradually become more common (P<0.001). Lunch breaks per week decreased from 5 in 2017 to 4 in 2019 (P<0.001). Trainees’ sense of educational deprivation due to physician assistants increased from 17.5% in 2016 to 25.6% in 2018 (P<0.001). Awareness of tasks and program/work achievement goals increased from 29.2% in 2016 to 58.3% in 2018 (P<0.001). Satisfaction with the learning environment increased over time, whereas satisfaction with working conditions varied.

**Conclusion:**

The Medical Resident Act has brought promising changes to the training of medical residents in Korea, as well as their satisfaction with the training environment.

## Introduction

### Background/rationale

Issues regarding the training environment of interns and residents who care for patients on the front line have regularly been raised. Previous research has found that residents’ training environment affects the safety of patients [1,2]. Out of a total of 105,628 physicians in South Korea, 13,636 (12.9%) trainees worked in medical institutions in 2019 [3]. In Korea, interns circulate across various departments for a year, and residents work to develop knowledge and skills in a specific field for 3 or 4 years. To contribute to securing patient safety and fostering excellent medical personnel by protecting the rights of residents, the Korean government legislated the Act for the Improvement of Training Conditions and Status of Medical Residents (Medical Resident Act) on December 3, 2015 based on a proposal made by Dr. Yong-ik Kim, a member of the National Assembly of Korea at that time [4]. This law was anticipated to improve the educational conditions of resident physicians and to provide a more favorable environment for high-quality resident training [5,6]. The Medical Resident Act and its relevant regulations specifically describe training conditions in terms of the curriculum, breaks, training programs, and remuneration, mainly covering the working environment (work hours of no more than 80 hours per week on average for 4 weeks, extended shifts of no more than 36 hours, and at least 10 hours of rest after an extended shift exceeding 16 hours) and the learning environment (designation and role of attending educators, development of training achievement goals, and annual assessment of training conditions). After 1 preparatory year, the Medical Resident Act was implemented at all training institutions, in all matters except for the work hour regulations, for which another preparatory year was allowed.

Historically, in the United States, the Libby Zion case helped alter work hour limitations and augmented supervisory requirements in residency training [7]. In the United States, the ACGME sought to improve health care by evaluating and improving the quality of residents' education through exemplary accreditation. Specifically, they emphasized limited duty hours, which averaged 80 hours per week [8]. The European Union has exempted the rules of working hours for trainees and now applies the same standard of up to 48 hours per week [9].

After the implementation of the Medical Resident Act, the Korean Intern Resident Association (KIRA) designed a project to check whether the working hours and training environment changed after the implementation of the Medical Resident Act. Therefore, nationwide intern-resident surveys were conducted starting in 2016.

### Objectives

This study aimed to analyze the working and training environment of residents in South Korea before and after the implementation of the Medical Resident Act in 2017. Although this Act was enacted in December 2015, there was a grace period for enforcement. This study also investigated residents' satisfaction with their working hours and educational programs. Specifically, information was collected every year by surveys administered to residents from 2016 to 2019 on working conditions, learning environment, job satisfaction, and satisfaction with the learning environment. Working conditions included consecutive work shifts, rest after a consecutive shift (hours), average sleep hours daily in the previous week (hours), number of lunch breaks in the previous week, 24-hour periods without duty in the last 4 weeks (times/wk), vacation days actually taken last year (days), admitted patient load during regular shifts (number). and admitted patient load during consecutive shifts (number). The working burden of internal medicine and general surgery residents was added. The learning environment included the awareness of learning achievement goals (%), sufficient training for attending educators (Likert score), and confidence in practicing without supervision after training. Other aspects of the learning environment included non-essential work as a resident, the involvement of physician assistants (PAs), and a sense of educational deprivation due to PAs. Satisfaction with the working and learning environment included general satisfaction.

## Methods

### Ethics statement

As this study used anonymized secondary data, the Institutional Review Board of Korea University College of Medicine, after reviewing the concept and design of the study, waived the need for ethical approval (KUIRB-2019-0241).

### Study design

This study presents the results of an annual cross-sectional survey study performed by the KIRA.

### Setting

The surveys were done between September and October from 2016 to 2019. Survey questionnaires were dispatched to the subjects through email. Subjects responded on the website provided by the KIRA. The somewhat different questionnaire items were used each year. Data from respondents were collected and saved for analysis. Some of the same interns or residents were able to participate in the survey each year. However, due to the anonymity of the respondents, it was not possible to trace the responses of specific individuals.

### Participants

Members of the KIRA, who were physicians enrolled in training programs run by accredited educational institutions in Korea (i.e., interns [postgraduate year 1, PGY 1] and residents [postgraduate years 2, 3, 4, or 5 [PGY 2, 3, 4, 5]) were invited to participate in the surveys (Table 1). There were no inclusion or exclusion criteria. All interns and residents were targeted for participation.

### Data sources/measurement

The survey questionnaire is available in Supplement 1. The survey items consisted of ordinal data of a 5-point Likert type scale (1, strongly disagree; 5, strongly agree) or binary nominal data. Among the original survey questions, we analyzed items that met the following 2 selecting conditons. First, items providing information about the working and learning environment of interns and residents were analyzed, as described in Tables 2–5. Second, the questionnaires of each year were different; therefore, items with a common meaning were selected for comparisons between years. The coding manual in Supplement 2 shows how the common items were matched.

### Items of the measurement tool (survey questionnaire)

#### Definition of training environment attributes

We analyzed questions that elicited information on attributes of the training environment for interns and residents, such as working hours, vacations, learning opportunities, and wellness.

#### Classification of institutions by type

Training hospitals and institutions were classified into 3 categories, as follows. Group A included 42 tertiary hospitals specializing in training, research, and critical patient care. Group B included other training hospitals with 100 or more physicians under training. Group C included the remaining general hospitals, comprising single-specialty hospitals such as ophthalmic or psychiatric hospitals, and other accredited organizations such as medical schools or research institutes with residency training programs.

#### Grouping of specialties

All 26 specialties currently accredited in Korea were regrouped into 5 categories in terms of similarities in a training context, such as workload or night shifts: internal medicine; general surgery; medical specialties other than internal medicine (pediatrics, neurology, psychiatry, and dermatology); surgical specialties other than general surgery (orthopedics, neurosurgery, plastic surgery, cardiothoracic surgery, ophthalmology, obstetrics and gynecology, and urology); and others (radiology, radiation oncology, nuclear medicine, laboratory medicine, preventive medicine, anesthesiology, rehabilitation, family medicine, and emergency medicine). Interns rotating within different specialties were grouped separately.

#### Working conditions

Lunch breaks, sleep time, the number of working hours throughout the day, and break hours were considered as part of training conditions. Weekly work hours, consecutive work shifts, and the working hours of each consecutive work shift were included. A consecutive work shift was considered a shift that included additional consecutive hours, such as requiring more consecutive work or work in the evening or on a holiday. Residents engaged in internal medicine and general surgery also provided the number of patients managed per day.

#### Learning environment

The learning environment included awareness of the achievement goals for training that are required to be presented to interns and residents (specified by specialty and year); whether the duties organized for trainees were actually related to those goals; and whether the trainees learned from the attending educators responsible for supervising them. As additional factors related to effective learning, non-physician duties, such as administrative paperwork, personal errands, and the activities of PAs, were considered. Satisfaction with working conditions and learning environment included general satisfaction.

### Validity and reliability testing

The content validity was discussed by the executive members of the KIRA, including the co-authors, before making the questionnaires. They agreed that the items sufficed to evaluate changes in residents’ working conditions, learning environment, and satisfaction. Reliability testing was not done because the items consisted of a variable format, including Likert-scale items, binary items, and nominal items.

### Bias

The proportion of respondents ranged from 20.8% to 34.5%. Therefore, there may have been response bias, although the number of responses was large.

### Study size

A *post hoc* analysis using G*Power (Heinrich-Heine-Universität Düsseldorf, Düsseldorf, Germany; http://www.gpower.hhu.de/) showed that the power was 0.99 when the variables with the least number of cells (124 and 134) were compared with an effect size of 0.5 and alpha error probability of 0.05 [10].

### Statistical methods

The outcomes of the annual survey among years were compared using the Kruskal-Wallis test. The Wilcoxon rank-sum test with the Bonferroni correction was performed for multiple comparisons between years. The Jonckheere-Terpstra test for 5-point Likert scale data and the Cochran-Armitage test for categorical data were used to identify statistically significant trends according to the year. All statistical analyses were conducted using IBM SPSS Statistics ver. 25.0 (IBM Corp., Armonk, NY, USA).

## Results

Raw data of the responses without the identification of individual training institutions is available from Dataset 1. Statistical analysis of results is available from Supplement 3.

### Participants

Of the total of 55,727 trainees enrolled in the KIRA, 15,029 interns and residents took part in the survey, and the number of survey participants increased year by year (from 2,984 in 2016 to 4,700 in 2019). Most of the participants worked in a tertiary hospital (67.0%–75.0%), and participants who responded to the survey had a similar distribution of characteristics among institutions and specialties (Table 1).

### Changes in working hours

**Fig. 1.**
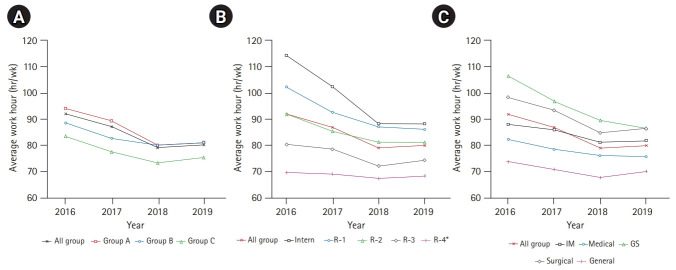
Weekly work hours of training physicians by institution, postgraduate year, and specialty. (A) By institution type (P-trend <0.01). (B) By postgraduate year (P-trend <0.01 except for *: P-trend=0.56). (C) By specialty (P-trend <0.01). Statistical significance was calculated using the Jonckheere-Terpstra test. Group A: tertiary hospitals; group B: general hospitals with 100 or more training physicians; group C: general hospitals with <100 training physicians, single-specialty hospitals, or other teaching institutions not classified in groups A or B. R, residency year; IM, internal medicine; GS, general surgery.

Although 23.9% of physicians trained in 2019 said they still worked continuously for more than 36 hours, they had breaks of 10 hours or more between such shifts; these breaks have gradually become more common. However, the number of lunch breaks per week decreased from 5 in 2017 to 4 in 2019. Across institutions, postgraduate years, and specializations, trainees worked overtime (90 hours a week) prior to the enforcement of the Medical Resident Act. The overall working hours tended to decrease, although interns worked the most (P<0.001, 114 hours in 2016, 88 hours in 2019) (Table 2, Fig. 1)

### Change in the workload of internal medicine and general surgery residents

Residents were asked about the number of patients admitted during regular shifts and the number of patients admitted during extended shifts, and the results showed that the workload did not change (Table 3). However, internal medicine residents showed a decreased workload during extended shifts.

### Changes in the learning environment

After the enforcement of the Medical Resident Act, awareness of tasks or program/work achievement goals significantly increased among interns and residents, especially interns (P<0.001, 29.2% in 2016 to 58.3% in 2018) (Table 4). Higher scores were given for the establishment of training programs to achieve these goals year by year, reflecting the gradual preparation of attending educators for sufficient training. In terms of current specialization training, trainees showed significantly positive responses regarding their future professional skills.

### Non-essential physician tasks and sense of educational deprivation due to the institution’s PAs

Non-essential physician tasks such as administrative paperwork or personal errands remained steady among interns—25.8% in 2016 versus 23.8% in 2019 (P<0.001) (Table 5). Although the number of institutions where PAs are involved in medical practice decreased, trainees’ overall sense of educational deprivation due to the institution’s employment of PAs increased from 17.5% in 2016 to 25.6% in 2018.

### Satisfaction with the working environment

**Fig. 2.**
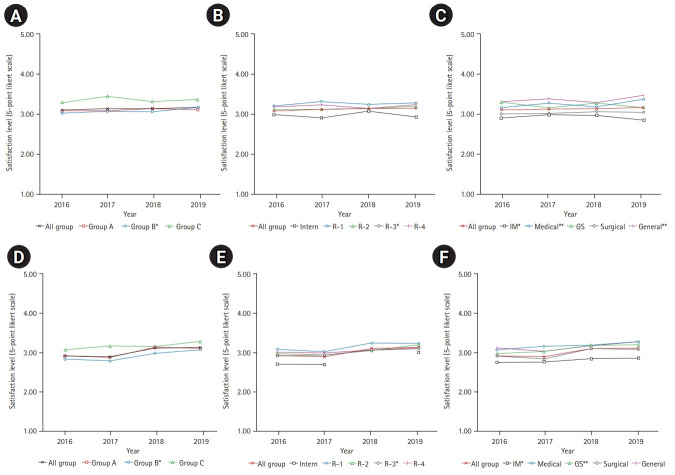
Overall satisfaction with the working and training environment among training physicians during 2016–2019. (A–C) Working environment. (D–F) Learning environment. (A) By institution type (*P-trend=0.02). (B) By postgraduate year (*P-trend=0.02). (C) By specialty (*P-trend=0.046, **P-trend <0.01). (D) By institution type (P-trend <0.01). (E) By postgraduate year (P-trend <0.01 except for *P-trend=0.02). (F) By specialty (data for interns in 2018 were not assessed; P-trend <0.01 except for *P-trend=0.02 and **P-trend=0.01). Statistical significance was calculated using the Jonckheere-Terpstra test. Group A: tertiary hospitals; group B: general hospitals with 100 or more training physicians; group C: general hospitals with <100 training physicians, single-specialty hospitals, or other teaching institutions not classified in groups A or B. R, residency year; IM, internal medicine; GS, general surgery.

The changes from 2016 to 2019 are presented in Fig. 2A–C. Satisfaction with the working environment varied according to the type of institution, postgraduate year, and specialty.

### Satisfaction with the learning environment

Satisfaction with the learning environment generally increased year by year from 2016 to 2019 for all types of institutions, postgraduate years, and specialties (Fig. 2D–F).

## Discussion

### Key results

The weekly working hours of residents decreased year by year. Interns worked the most (114 hours in 2016, 88 hours in 2019). It became increasingly common for residents to have 10 hours or more of break time. The frequency of having lunch breaks per week decreased from 5 in 2017 to 4 in 2019. Residents’ sense of educational deprivation due to PAs increased from 17.5% in 2016 to 25.6% in 2018. There was a more favorable learning environment. For example, residents’ awareness of tasks or program/work achievement goals increased from 29.2% in 2016 to 58.3% in 2018. Although residents’ satisfaction with working conditions varied, their satisfaction with the learning environment increased year by year.

### Interpretation

Prior to the Medical Resident Act, intern and resident education was burdensome to the health and wellbeing of residents. However, within the 2-year period after the implementation of the Medical Resident Act, training institutions began to make changes to meet the new standards. Weekly work hours for training physicians decreased to 80.2 hours, and they were provided with more full days off-duty as well as guaranteed vacations (Table 2). The role of attending educators was better recognized by training physicians, who also reported gaining more confidence in their competencies in practicing medicine on their own (Table 4). However, reduced working hours resulted in increased work pressure, leading to a lack of lunch breaks or excessive patient loads during night shifts (Table 2). The activities of PAs were increasingly regarded as interfering with residents’ learning opportunities (Table 5). These overall changes appeared to have a limited effect on interns (Table 2).

Ensuring duty hour restrictions was a major aim of the Medical Resident Act and a major controversial issue. With the passing of the legislation, concerns were raised that it would be too difficult to reduce training physicians’ working hours given practical circumstances, such as general hospitals with an excessive patient load or smaller institutions with chronic workforce shortages [11]. However, the above results showed that working conditions gradually began to comply with the new standards, with more full days off-duty and guaranteed vacation time.

Despite some promising changes under the Medical Resident Act, the findings of this study showed that more than 20% of residents still experienced consecutive shifts exceeding 36 continuous hours. The residents’ high labor intensity for patient care significantly increased after the Medical Resident Act to the point that some residents now skip lunch (Table 2).

The Medical Resident Act also brought about changes in the learning environment. Residents became more aware of what they should achieve throughout the program, rather than merely serving as an assistant workforce (Table 4). Such awareness may in turn have motivated the various stakeholders involved in teaching, including training institutions and attending educators. This may have led to greater appreciation among residents concerning both their programs and their teachers.

### Comparison with previous studies

A previous study compared the training environment between 2015 (1,793 people) and 2017 (1,768 people) before and after enactment of the Medical Resident Act. Residents worked more than 80 hours per week on average (2015, 92.4 hours; 2017, 87.3 hours). They worked twice as long as 36 hours in a consecutive shift (2015, 89.4 hours; 2017, 70.1 hours) [1,2]. According to the Medical Resident Act’s implementation in 2017, the Korean Society of Internal Medicine reorganized the training process based on competence. It strengthened the evaluation of training hospitals to overcome and normalize the crisis of internal medicine residency training [12]. Residents and faculty members of Chonnam National University Hospital agreed to the duty hour regulations of the Medical Resident Act, including a maximum duty of 80 hours per week (averaged over 4 weeks), a maximum consecutive shift length of less than 36 hours, and mandatory 10-hour breaks between shifts [13]. A survey of emergency medicine residents from 80 out of 97 training hospitals in 2013 showed that the average number of duty hours was 63.7±10.7 hours per week. Residents at 9 hospitals (11.3%) worked more than 80 hours per week on average [14]. According to a survey of 513 psychiatry residents in 2016 and 2017, the average working hours were distributed as follows: 35 (6.9%) worked for less than 9 hours; 188 (37.2%) worked for 9 to 11 hours; 185 (36.6%) worked for 11 to 13 hours; 57 (11.3%) worked for 13 to 15 hours; and 41 (8.1%) worked for more than 15 hours [15]. The above-described previous studies show the high labor intensity before implementation of the Medical Resident Act.

### Recommendations for improvement of residents’ working and learning environment

Despite these promising results, further improvement is required. Graduate medical education in Korea remains process-oriented, focusing on the number of cases diagnosed, reports published, or conferences attended as the main trainee goals. Moreover, some specialties simply repeat the same goals for each training year, lacking milestones or evaluation tools. Furthermore, our study showed that the proportion of residents who reported non-essential work remained approximately 18%, which could be readily reduced with clearer learning objectives.

It is of concern that, in addition to reduced training hours, more interns and residents felt deprived of learning opportunities by PAs. Unlike in countries where PAs are regarded as a distinct profession, the members of which individually and actively practice medicine to certain extent, boundaries between different healthcare domains (e.g., physicians, nurses, radiologists, etc.) are strictly enforced in Korea; thus, only physicians are permitted to perform medical procedures or engage in certain types of clinical decision-making in patient care, such as prescribing medications, using ultrasonography, or performing surgery, whereas PAs are unlicensed to do so. It seems that using PAs may be a more attractive alternative after the Medical Resident Act from an administrative perspective, but further research is needed on its effects in terms of teaching and learning.

### Limitation

The number of participants surveyed comprised approximately 20% of the total number of resident physicians and interns each year (more specific numbers are shown in Table 1). The small proportion of survey participants and the voluntary nature of responding to the survey imposed selection bias as a limitation of the study. Another limitation is the lack of knowledge of the details related to the current Act since the survey was commenced just before the enactment of the Act. Therefore, this study could not directly evaluate the degree of implementation of the Medical Resident Act. Based on the sample characteristics, with an apparent increasing trend over time, it appears that some of the same respondents repeated the survey across different years. Therefore, the samples in the different years were not independent. However, due to the anonymity of the survey, it was not possible to pair the responses from different years. The weakest point of these consecutive survey results originated from the use of different survey items each year. Therefore, only items with the same content were selected and compared. Some data were also missing from the 2019 survey.

### Suggestion

Further studies should include residents in other specialties, which are observed less frequently, and people working in small institutions with chronic labor shortages. In the future, it will be necessary to conduct a detailed evaluation according to the Medical Resident Act for matters regarding exhaustion, sleep, and other issues related to the quality of training. The results would also be more valuable if questionnaires were also administered to evaluators. For the future success of the Medical Resident Act in Korea, the government and medical societies must provide political and financial support. In addition to facilitating the development of an outcome-based curriculum, it is also necessary to expand the hospital system to address problems related to the shortage of physicians and the more efficient allocation of patients within each hospital.

### Conclusion

Significant changes have been made to residents’ working and learning environment after the legislation of the Medical Resident Act in Korea. To improve the quality of training, the Medical Resident Act in Korea attempted to reform training standards regarding the working and learning environments, and the results were successful.

## Figures and Tables

**Table 1.** Characteristics of the participants in the national intern-resident survey (2016–2019) in Korea

Characteristic	Year			
	2016	2017	2018	2019
Total PGYs per year	14,370	13,954	13,767	13,636
No. of respondents (%)	2,984 (20.8)	3,566 (25.6)	3,779 (27.5)	4,700 (34.5)
Institution type (%)				
Group A	2,239 (75.0)	2,628 (73.7)	2,739 (72.5)	3,150 (67.0)
Group B	344 (11.5)	492 (13.8)	553 (14.6)	750 (16.0)
Group C	401 (13.4)	446 (12.5)	487 (12.9)	800 (17.0)
Postgraduate year (%)				
Intern (PGY-1)	709 (23.8)	955 (26.8)	911 (24.1)	1108 (23.6)
R-1 (PGY-2)	472 (15.8)	633 (17.8)	642 (17.0)	873 (18.6)
R-2 (PGY-3)	566 (19.0)	640 (18.0)	647 (17.1)	870 (18.5)
R-3 (PGY-4)	621 (20.8)	711 (19.9)	803 (21.3)	979 (20.8)
R-4 (PGY-5)	616 (20.6)	627 (17.6)	776 (20.5)	870 (18.5)
Specialty (%)				
Internal medicine	442 (19.4)	461 (17.7)	481 (16.8)	643 (17.9)
Medical specialties other than internal medicine	397 (17.5)	453 (17.4)	547 (19.1)	673 (18.7)
General surgery	133 (5.9)	136 (5.2)	124 (4.3)	180 (5.0)
Surgical specialties other than general surgery	467 (20.5)	605 (23.2)	633 (22.1)	744 (20.7)
Other areas	836 (36.8)	955 (36.6)	1083 (37.8)	1352 (37.6)

Group A: tertiary hospitals; group B: general hospitals with 100 or more training physicians; group C: general hospitals with <100 training physicians, single-specialty hospitals, or other teaching institutions not classified in groups A or B. PGY, postgraduate year; R, residency year.

**Table 2.** Changes in trainees’ working hours

Variable	Year				P-value
	2016	2017	2018	2019	
Consecutive work shifts (no./wk)					
Internal medicine	2 (1–3)	2 (1–3)	2 (1–2)	NA	0.34
Medical specialties other than internal medicine	2 (0–3)	2 (1–3)	2 (1–3)	NA	0.19
General surgery	3 (2–3)^a^	2 (2–3)^b^	2 (2–2.5)^b^	NA	<0.001
Surgical specialties other than general surgery	2 (1–3)^a^	2 (1–3)^a,b^	2 (2–3)^b^	NA	<0.001
Experienced over 36 hours of continuous shift work in the last 4 weeks (%)	1,026/2,984 (34.4)	899/2,801 (32.1)	1,291/3,779 (34.2)	1,123/4,700 (23.9)	<0.001^a)^
Rest after a consecutive shift work (hr)					
Internal medicine	0 (0–6)^a^	0 (0–10)^b^	1.5 (0–11)^c^	10 (8–12)^d^	<0.001
Medical	0 (0–12)^a^	3.3 (0–12)^b^	4 (0–12)^b^	12 (9–13)^c^	<0.001
General surgery	0 (0–8)^a^	0 (0–10)^a^	0 (0–10)^a^	10 (8–12)^b^	<0.001
Surgical	0 (0–6)^a^	1 (0–10)^b^	5 (0–10)^b^	10 (6–12)^c^	<0.001
Average daily sleep hours in the previous week (hr)	6 (5–6)^a^	6 (5–6)^a^	6 (5–6)^b^	NA	<0.001
Lunch breaks last week (time/wk)	NA	5 (4–7)^a^	4 (2–5)^b,c^	4 (2–5)^c^	<0.001
24-hour period off duty in the last 4 weeks (times/wk)	4 (1–6)^a^	4 (2–6)^b^	4 (4–6)^c^	NA	<0.001
Vacation days actually taken last year (day)	10 (7–14)^a^	10 (10–14)^b^	14 (10–14)^c^	11 (10–14)^d^	<0.001

Values are presented as median (interquartile range) considering the skewness of the data distribution or number (%), unless otherwise stated. Comparisons are shown between years (^‘a’^, ^‘b’^, ^‘c’^, and ^‘d’^ denote no statistical significance if they are the same letter, and show statistical significance if they are different letters). NA, not assessed. Statistical significance was calculated using a)the Cochran–Armitage test or the Kruskal-Wallis test and Wilcoxon rank-sum test with the Bonferroni correction.

**Table 3.** Number of patients admitted during regular shifts and the number of patients admitted during extended shifts of internal medicine and general surgery residents from 2016 to 2019

Variable	Year				P-value
	2016	2017	2018	2019	
Admitted patient load during regular shifts (no.)	NA				
Internal medicine		20 (20–27)	20 (18–25)	20 (18–25)	0.84
General surgery		25 (18–30)	25 (20–30)	20 (15–30)	0.07
Admitted patient load during extended shifts (no.)	NA				
Internal medicine		90 (50–120)^a^	120 (80–150)^b^	100 (70–150)^b^	<0.001
General surgery		80 (50–100)^a^	90 (70–120)^b^	80 (60–100)^a,b^	0.03

Values are presented as median (interquartile range) considering the skewness of the data distribution. Statistical significance was calculated using the Kruskal-Wallis test and Wilcoxon rank-sum test with the Bonferroni correction. Comparisons are shown between years (^‘a’^, ^‘b’^, ^‘c’^, and ^‘d’^ denote no statistical significance if they are the same letter, and show statistical significance if they are different letters). NA, not assessed.

**Table 4.** Aspects of the learning environment among residents from 2016 to 2019 in Korea

Variable	Year				P-value
	2016	2017	2018	2019	
Awareness of training achievement goals (%)	1,179/2,189 (53.9)	952/1,378 (69.1)	1,843/2,589 (71.2)	NA	<0.001^a)^
Interns (PGY 1)	161/551 (29.2)	14/32 (43.8)	333/571 (58.3)		<0.001^a)^
Residents (PGY 2–5)	1,018/1,638 (62.2)	938/1,346 (69.7)	1,510/2,018 (74.8)		<0.001^a)^
Establishment of a training program for each goal (Likert score)	3 (2–4)^a^	3 (2–4)^b^	3 (3–4)^b^	4 (3–4)^c^	<0.001
Group A	3 (2–4)^a^	3 (2–4)^b^	3 (3–4)^b^	4 (3–4)^c^	<0.001
Group B	3 (2–4)^a^	3 (2–4)^a,b^	3 (2–4)^b^	3 (3–4)^c^	<0.001
Group C	3 (2–4)^a^	3 (2–4)^a,b^	3 (3–4)^b^	4 (3–4)^c^	<0.001
Providing sufficient training for attending educators (Likert score)	3 (2–4)^a^	3 (2–4)^a^	3 (3–4)^b^	3 (3–4)^b^	<0.001
Confidence in practicing the specialty without supervision after training	3 (2–4)^a^	3 (2–4)^b^	3 (3–4)^c^	3 (3–4)^d^	<0.001

Values are presented as number (%) or median (interquartile range) considering the skewness of the data distribution, unless otherwise stated. Group A: tertiary hospitals; group B: general hospitals with 100 or more training physicians; group C: general hospitals with less than 100 training physicians, single-specialty hospitals, or other teaching institutions not classified in groups A or B. Comparisons are shown between years (^‘a’^, ^‘b’^, ^‘c’^, and ^‘d’^ denote no statistical significance if they are the same letter, and show statistical significance if they are different letters). NA, not assessed; PGY, postgraduate year. Statistical significance was calculated using ^a)^the Cochran–Armitage test or the Kruskal-Wallis test and Wilcoxon rank-sum test with the Bonferroni correction.

**Table 5.** Trends in non-essential physician tasks and sense of educational deprivation due to the institution’s use of PAs from 2016 to 2019

Variable	Year				P-value
	2016	2017	2018	2019	
Non-essential physician tasks (%)	18.5	18.5	22.6	18.1	<0.001
Interns (PGY 1)	25.8	25.7	29.4	23.8	<0.001
Residents (PGY 2–5)	16.3	15.9	20.4	16.4	<0.001
Involvement of PAs in medical practice (no., %)	2,617/2,984 (87.7)	1,992/2,606 (76.4)	2,821/3,779 (74.7)	NA	<0.001
Group A	1,991/2,239 (88.9)	1,491/1,930 (77.3)	2,025/2,739 (73.9)		<0.001
Group B	295/344 (85.8)	273/350 (78)	440/553 (79.6)		0.02
Group C	331/401 (82.5)	228/326 (69.9)	356/487 (73.1)		<0.001
Educational deprivation sense due to the institution’s PA: yes (no., %)	523/2,984 (17.5)	495/1,970 (25.1)	722/2,821 (25.6)	NA	<0.001
Group A	421/2,239 (18.8)	379/1,476 (25.7)	508/2,025 (25.1)		<0.001
Group B	50/344 (14.5)	65/269 (24.2)	117/440 (26.6)		<0.001
Group C	52/401 (13)	51/225 (22.7)	97/356 (27.3)		<0.001

Group A: tertiary hospitals; group B: general hospitals with 100 or more training physicians; group C: general hospitals with <100 training physicians, single-specialty hospitals, or other teaching institutions not classified in groups A or B. Statistical significance was calculated using the Cochran–Armitage test or the Jonckheere-Terpstra test. PA, physician assistant; PGY, postgraduate year; NA, not assessed.

## Footnotes

**Data availability** Data files are available from Harvard Dataverse: https://doi.org/10.7910/DVN/QWXJJE Dataset 1. Raw data of the responses without the identification of individual training institutions.

## Supplementary materials

Supplementary files are available from Harvard Dataverse: https://doi.org/10.7910/DVN/QWXJJE

Supplement 1. Survey questionnaires of each year in Korean.

Supplement 2. Coding manual for the analysis of raw data.

Supplement 3. Statistical analysis results.

Supplement 4. Audio recording of the abstract.

## References

[1] Oh SH, Kim JS (2019). Changes in the training conditions of residents by enforcement of medical residents Act. J Digit Converg.

[2] Oh SH, Kim JS, Lee PS (2015). A survey on training and working conditions of residents in 2015. J Korean Med Assoc.

[3] Statistics Korea (2019). Status of medical resource by province in Korea [Internet]. https://kosis.kr/statHtml/statHtml.do?orgId=350&tblId=TX_35003_A003&conn_path=I3.

[4] Huh S (2016). Will the year 2016 augur well for better patient safety and health of residents in Korea according to the enactment of the Act for improving the resident training environment and enhancing resident’s status?. J Educ Eval Health Prof.

[5] (2015). Act for The Improvement of Training Conditions and Status of Medical Residents, Act No. 13600 (Dec 22, 2015) [Internet]. https://www.law.go.kr/LSW/eng/engLsSc.do?menuId=2&section=lawNm&query=medical+resident+act&x=0&y=0#liBgcolor0.

[6] (2019). Act for The Improvement of Training Conditions and Status of Medical Residents, Act No. 16260 (Jan 15, 2019) [Internet]. https://www.law.go.kr/LSW/eng/engLsSc.do?menuId=2&section=lawNm&query=medical+resident+act&x=0&y=0#liBgcolor0.

[7] Asch DA, Parker RM (1988). The Libby Zion case: one step forward or two steps backward?. N Engl J Med.

[8] Wolf SJ, Akhtar S, Gross E, Barnes D, Epter M, Fisher J, Moreira M, Smith M, House H (2018). ACGME clinical and educational work hour standards: perspectives and recommendations from emergency medicine educators. West J Emerg Med.

[9] (2000). Directive 2000/34/EC of the European Parliament and of the Council of 22 June 2000 amending Council Directive 93/104/EC concerning certain aspects of the organisation of working time to cover sectors and activities excluded from that Directive [Internet]. http://data.europa.eu/eli/dir/2000/34/oj.

[10] Faul F, Erdfelder E, Lang AG, Buchner A (2007). G*Power 3: a flexible statistical power analysis program for the social, behavioral, and biomedical sciences. Behav Res Methods.

[11] Lee W, Kim SY, Lee SI, Lee SG, Kim HC, Kim I (2018). Barriers to reporting of patient safety incidents in tertiary hospitals: a qualitative study of nurses and resident physicians in South Korea. Int J Health Plann Manage.

[12] Eom JS (2018). Changes in regulation of internal medicine residency training and evaluation of teaching hospitals. Korean J Med.

[13] Han ER, Chung EK (2020). The perception of medical residents and faculty members on resident duty hour regulation. Korean J Med Educ.

[14] Chung SP, Kang HG, Kim HJ, Ryu JH, Park YS, Seo DW, Yoon YH, Yoon JC, Lee K, Lee JY, Jeung KW (2014). Current duty hours of emergency resident physicians in Korea: multicenter cross-sectional study. J Korean Soc Emerg Med.

[15] Kim JH, Kyeon YG, Kim JW, Oh HS, Lee SM, Seo JS, Jung SW (2019). Survey on the environment and condition of Korean psychiatric residents from 2016 to 2017 years. J Korean Neuropsychiatr Assoc.

