# Supplementary material for: Changes in the working conditions and learning environment of medical residents after the enactment of the Medical Resident Act in Korea in 2015: a national 4-year longitudinal study
Source: J Educ Eval Health Prof. 2021 Apr 20;18:7. doi: 10.3352/jeehp.2021.18.7 (PMC8118751; doi:10.3352/jeehp.2021.18.7)
Supplement: Supplementary file 2 — Supplement 1. Survey questionnaires of each year in Korean. [file jeehp-18-07-suppl1.docx]

**2016년 전공의 수련병원 평가 설문조사**

안녕하십니까?

대한전공의협의회는 전국 수련병원에서 일하는 전공의들이 직접 참여하는 전국 수련병원 평가 설문조사를 실시하고 있습니다.

본 설문의 문항은 현재 전공의들의 근무환경 및 교육환경에 관한 것으로 검증된 자료를 기초하여 선정하였습니다. 설문 결과는 추후 대한전공의협의회가 진행할 **수련환경 개선을 위한 자료, 전국 수련병원 평가 사이트 “닥터브릿지”**에 활용할 예정입니다.

조사결과는 통계법 제33조 2항에 의거하여, 통계의 작성을 위하여 수집된 개인이나 법인 또는 단체 등의 비밀에 속하는 자료는 통계작성 외의 목적으로 사용되지 않을 것입니다. 귀중한 시간을 내시어 응답해 주시면 진심으로 감사드립니다.

* 본 설문조사는 응답자 여러분의 참여에 대한 감사의 표시로 참가자들에게 추첨을 통한 경품 이벤트를 준비했습니다. 본인의 연락처를 적어주시면 감사하겠습니다.

| **- 전체 응답자 중 추첨을 통해 상품 증정**  **1등 : 삼성 갤럭시 탭프로S (1명)**  **2등 : 백화점 상품권 5만원 (10명)**  **3등 : 아이스 아메리카노 (50명)** |
| --- |

설문조사 기간 : 2016년 8월 12일 ~ 2016년 9월 9일

담당 : 대한전공의협의회 사무국

전화 : 02-796-6127, 6128 e-mail : office@youngmd.org

**< 개인 정보 입력 >**

1. 귀하의 성별을 다음의 보기에서 선택해 주시기 바랍니다.

1) 남 2) 여

2. 귀하의 수련병원은?

3. 귀하의 수련 연차를 다음의 보기에서 선택해 주시기 바랍니다.

1) 인턴 2) 레지던트 1년 3) 레지던트 2년 4) 레지던트 3년 5) 레지던트 4년

4. 귀하의 수련과를 다음의 보기에서 선택해 주시기 바랍니다.

(레지던트 선생님만 선택해주세요)

1) 가정의학과 2) 결핵과 3) 내과 4) 마취통증의학과 5) 방사선종양학과

6) 병리과 7) 비뇨기과 8) 산부인과 9) 성형외과 10) 소아청소년과

11) 신경과 12) 신경외과 13) 안과 14) 영상의학과 15) 외과

16) 응급의학과 17) 이비인후과 18) 재활의학과 19) 정신과 20) 정형외과 21) 직업환경의학과 22) 진단검사의학과 23) 피부과 24) 핵의학과 25) 흉부외과 26) 예방의학과 27) 기타 ( )

5. 본인의 연락처(핸드폰 번호)를 적어주십시오. (경품추첨용도, 공란 시 추첨 제외)

☎

**< 객관적 평가 항목 >**

6. 1주일에 평균 몇 시간 근무하십니까? 근무시간은 병원에 있었던 총 시간으로 계산합니다. (1주일은 총 168시간입니다.)

( ) 시간/1주일

7. 귀하는 지난 1주일간 최대 연속 수련시간이 36시간을 초과한 적이 있습니까?

1) 예 2) 아니오

8. 지난 1주일 간 귀하의 하루 평균 수면시간은 몇 시간 이었습니까? (지난 1주일이 휴가 등 특별한 기간이었을 경우, 그 전 1주일에 대하여 답해주시기 바랍니다.)

( ) 시간/1일

9. 한 끼 식사 시에 사용할 수 있는 실질적인 시간(식사시간 + 휴게시간)은 얼마나 되십니까?

( 　）분

10. 당직 근무(24시간 근무 or 야간근무)를 주 몇 회나 하십니까 ?

( ) 회/1주

11. 당직 근무 종료 후 정규시작 전까지 실질적으로 휴게시간이 얼마나 보장되십니까?

( ) 시간

12. 1개월 근무 시 평균 휴일(off, 휴가 제외)은 며칠이나 부여받습니까?

( ) 일 /1개월

13. 지난해(2015년) 귀하께서 전공의(인턴, 레지던트)로 근무하셨다면 실제 휴가를 며칠 가셨습니까? (주말 및 공휴일 제외)

( ) 일

14. 귀하의 월 평균 실수령액은 얼마 입니까?

( ) 만원

15. 귀하의 평일 하루 평균 당직비는 얼마입니까 ?

평일 ( ) 만원/일

16. 귀하의 주말 하루 평균 당직비는 얼마입니까 ?

주말 ( ) 만원/일

17. 본인의 업무 중 전공의 수련과 관련 없는 업무가 차지하는 비중이 얼마나 되십니까?

(예시 : 의국 내 서류정리 등과 같이 행정직원이 대체 가능한 업무 또는 본인과 관련 없는 논문잡일 등)

( ) %

18. 주치의 역할을 하는 경우에 환자를 한 번에 평균 몇 명 담당합니까?

( ) 명

19. 귀하가 당직일 때 응급환자에 대한 논의가 필요한 경우 담당 교수와 신속하고 원활한 소통이 가능합니까?

1. 예 2. 아니오

20. 귀하는 교수 또는 상급 전공의에게 불쾌한 성희롱(말), 성추행(행동)을 당한 적이 있습니까?

1. 예 2. 아니오

21. 귀하는 교수 또는 상급 전공의에게 언어적, 신체적 폭력을 당한 적이 있습니까?

1. 예 2. 아니오

22. 귀하는 교수 또는 지도전문의로부터 논문을 이용한 협박(모든 형태)을 당한 적이 있습니까?

1. 예 2. 아니오

23. 귀하의 병원에 PA(Physician Assistant)가 있습니까?

1. 예 2. 아니오

* PA(Physician Assistant)는 합법적이지 않으나, 의사의 업무 중 일부를 위임받아 대신하는 간호사를 말하며, Surgery Assistant, Nurse Practitioner, Ulicensed Assistant 등 부르는 명칭이 다양하다.

24. 귀하의 병원에서 PA(Physician Assistant)가 의사만이 할 수 있는 술기를 직접 하는 것을 본 적이 있습니까?

1. 예 2. 아니오

25. 귀하의 병원에서 PA(Physician Assistant)가 의사만이 할 수 있는 약 처방을 직접 하는 것을 본 적이 있습니까?

1. 예 2. 아니오

26. 귀하의 병원에서 PA(Physician Assistant)가 의사만이 할 수 있는 수술을 직접 집도하는 것을 본 적이 있습니까?

1. 예 2. 아니오

27. 귀하께서 PA(Physician Assistant)로 인해 교육적 기회를 박탈당했다고 느낀 적이 있습니까?

1. 예 2. 아니오

**< 주관적 평가 항목 >**

28. 귀하가 근무하고 있는 기관의 **근무환경**을 평가해 주십시오.

|  | ◀그렇지 않다. 그렇다.▶  ① ② ③ ④ ⑤ |
| --- | --- |
| 1. 전반적인 근무환경에 만족한다. | ○ ○ ○ ○ ○ |
| 2. 근무 강도에 만족한다. | ○ ○ ○ ○ ○ |
| 3. 급여 지급 수준에 만족한다. | ○ ○ ○ ○ ○ |
| 4. 당직수당 및 기타상여금 지급 수준에 만족한다. | ○ ○ ○ ○ ○ |
| 5. 휴가(연가, 병가, 출산휴가 포함) 사용이 자유롭다. | ○ ○ ○ ○ ○ |
| 6. 결혼, 임신, 경조사 등 개인적인 사정에 대한 스케줄의 유연성이 있다. | ○ ○ ○ ○ ○ |
| 7. 근로 환경(당직실 포함) 및 전반적인 시설에 만족한다. | ○ ○ ○ ○ ○ |
| 8. 기타 전공의 복지 환경에 만족한다. | ○ ○ ○ ○ ○ |
| 9. 의료인 간의 뚜렷한 업무 분배가 되어 있다. | ○ ○ ○ ○ ○ |
| 10. 병원 내 사회생활에 있어, 상호간 인격 존중을 한다. | ○ ○ ○ ○ ○ |

29. 귀하가 근무하고 있는 기관의 **교육환경**을 평가해 주십시오.

|  | ◀그렇지 않다. 그렇다.▶  ① ② ③ ④ ⑤ |
| --- | --- |
| 1. 전반적인 교육환경에 만족한다. | ○ ○ ○ ○ ○ |
| 2. 환자군과 질환의 다양성이 있다. | ○ ○ ○ ○ ○ |
| 2. 임상능력 함양을 위한 참여 기회(시술 참여, 의사결정과정 참여 등)가 있다. | ○ ○ ○ ○ ○ |
| 3. 지도전문의에게 적절한 교육적 지도를 받고 있다. | ○ ○ ○ ○ ○ |
| 4. 집담회 등 학술활동이 수련과정에 충분히 포함되어 있다. | ○ ○ ○ ○ ○ |
| 5. 교육자료(학술논문, 인터넷 자료 등) 접근도에 만족한다. | ○ ○ ○ ○ ○ |
| 6. 해외 연수 및 학회 참여 기회에 만족한다. | ○ ○ ○ ○ ○ |
| 7. 타 수련병원에의 파견 수련에 만족한다. | ○ ○ ○ ○ ○ |
| 8. 연차별(인턴 포함)로 수련 받아야 할 항목이 제시되어 있다. | ○ ○ ○ ○ ○ |
| 9. 제시된 연차별 수련교육과정(인턴 포함)이 체계적 진행되고 있다. | ○ ○ ○ ○ ○ |
| 10. 현재 전공의 수련이 수련 직후 봉직을 하거나  개원하는데 충분하다. | ○ ○ ○ ○ ○ |

30. 대한전공의협의회에 건의사항이나 수련환경 개선을 위한 의견을 자유롭게 적어주세요.

**설문에 응해주셔서 대단히 감사합니다.**

**2017년 전국 전공의 병원평가 설문조사**

안녕하십니까?

대한전공의협의회는 전국 수련병원에서 일하는 전공의들이 직접 참여하는 **전국 전공의 병원평가 설문조사**를 실시하고 있습니다.

본 설문의 문항은 현재 전공의들의 근무환경 및 교육환경에 관한 것으로 검증된 자료를 기초하여 선정하였습니다. 설문 결과는 추후 대한전공의협의회가 진행할 **수련환경 개선을 위한 자료, 전국 수련병원 평가 사이트 “닥터브릿지”**에 활용할 예정입니다.

조사결과는 통계법 제33조 2항에 의거하여, 통계의 작성을 위하여 수집된 개인이나 법인 또는 단체 등의 비밀에 속하는 자료는 통계작성 외의 목적으로 사용되지 않을 것입니다. 귀중한 시간을 내시어 응답해 주시면 진심으로 감사드립니다.

* 본 설문조사는 응답자 여러분의 참여에 대한 감사의 표시로 참가자들에게 추첨을 통한 경품 이벤트를 준비했습니다. 본인의 연락처를 적어주시면 감사하겠습니다.

*** 설문에 응답할 때 “최근 1년 간” 기준으로 답해주기를 부탁드립니다.**

| **- 전체 응답자 중 추첨을 통해 상품 증정**  **1등 : 애플 아이패드미니 iPad Mini 4 Wi-Fi 128GB (2명)**  **2등 : 백화점 상품권 5만원 (10명)**  **3등 : 아이스 아메리카노 (50명)** |
| --- |

설문조사 기간 : 2017년 9월 29일 ~ 2017년 10월 31일

담당 : 대한전공의협의회 사무국

전화 : 02-796-6127, 6128 e-mail : office@youngmd.org

**< 개인 정보 입력 >**

1. 귀하의 성별을 다음의 보기에서 선택해 주시기 바랍니다.

1) 남 2) 여

2. 귀하의 수련병원은?

3. 귀하의 수련 연차를 다음의 보기에서 선택해 주시기 바랍니다.

1) 인턴 2) 레지던트 1년 3) 레지던트 2년 4) 레지던트 3년 5) 레지던트 4년

4. 귀하의 수련과를 다음의 보기에서 선택해 주시기 바랍니다.

1) 가정의학과 2) 결핵과 3) 내과 4) 마취통증의학과 5) 방사선종양학과

6) 병리과 7) 비뇨기과 8) 산부인과 9) 성형외과 10) 소아청소년과

11) 신경과 12) 신경외과 13) 안과 14) 영상의학과 15) 외과

16) 응급의학과 17) 이비인후과 18) 재활의학과 19) 정신건강의학과

20) 정형외과 21) 직업환경의학과 22) 진단검사의학과 23) 피부과

24) 핵의학과 25) 흉부외과 26) 예방의학과 27) 인턴 28) 기타 ( )

5. 본인의 연락처(핸드폰 번호)를 적어주십시오. **-> 필수응답 아님**

*경품 추첨에 참여하실 경우 핸드폰 번호가 필수 사항입니다.

1) 응모 안함

2) 응모 (010-0000-0000)

_____________________________________

**< 전공의 수련 환경 객관적 평가>**

6. 1주일에 평균 몇 시간 근무하십니까? 근무시간은 병원에 있었던 총 시간으로 계산합니다. (1주일은 총 168시간입니다.)

( ) 시간/1주일

7. 귀하는 지난 4주 동안 최대 연속 수련시간인 36시간을 초과한 적이 있습니까?

1) 예 2) 아니오

8. 1주일 간 귀하의 하루 평균 수면시간은 몇 시간 입니까?

( ) 시간/1일

9. 점심 식사시간에 식사를 할 수 있는 경우가 일주일에 몇 번이나 되십니까?

( 　）번

10. 한 끼 식사 시에 사용할 수 있는 실질적인 시간(식사시간 + 휴게시간)은 얼마나 되십니까?

( 　）분

11. 식사 시간에 받는 콜은 평균 몇 번 정도 입니까?

( 　）번

12. 야간이나 휴일에 당직근무(응급의학과의 경우 야간 근로)를 주 몇 회나 하십니까 ?

( ) 회/1주

13. 귀하가 지금까지 경험한 최대 연속 당직일수는 며칠입니까?

최대 ( )일

14. 당직 근무 종료 후 정규시작 전까지 실질적으로 휴게시간이 얼마나 보장되십니까?

( ) 시간

15. 1개월 근무 시 평균 휴일(24시간 출근하지 않는 날, 휴가 제외)은 며칠이나 부여받습니까?

※ 휴일(off): 연차휴가(14일)를 제외하고 24시간 연속으로 쉬는 날, 토일, 공휴일 포함

※ 휴일은 완전한 Duty-off로, house-call을 받고 나간 경우는 휴일에서 제외

( ) 일 /1개월

16. 지난해(2016년) 귀하께서 전공의(인턴, 레지던트)로 근무하셨다면 실제 휴가를 며칠 가셨습니까? (주말 및 공휴일 제외)

1. 해당 사항 없음 2. ( ) 일

17. 귀하의 연봉은 얼마입니까? (세후 기준)

( ) 만원

18. 귀하의 평일 하루 평균 당직비는 얼마입니까 ? (일단위로 환산하여 작성)

1. 평일 ( ) 만원/일 2. 해당 사항 없음 3. 모름

19. 귀하의 휴일 하루 평균 당직비는 얼마입니까 ? (일단위로 환산하여 작성)

1. 휴일 ( ) 만원/일 2. 해당 사항 없음 3. 모름

20. 귀하의 ‘정규’ 업무 중 전공의 수련과 관련 없는 업무가 차지하는 비중이 얼마나 되십니까? (예시 : 의국 내 서류정리 등과 같이 행정직원이 대체 가능한 업무 또는 본인과 관련 없는 논문잡일 등)

( ) %

21. 귀하는 전공의 선발과정(인턴의 경우, 인턴선발과정)이 공정하고 객관적으로 이루어진다고 생각하십니까?

1. 예 2. 아니오

22. 귀하는 인턴이나 레지던트를 처음 시작했을 때, 혹은 텀이 바뀌었을 때, 오프(off, 휴일)를 나가지 못하도록 강요 받았던 적이 있습니까?

1. 예 2. 아니오

23. 귀하는 징계 등의 목적으로 오프를 나가지 못했던 적이 있습니까?

1. 예 2. 아니오

24. 귀하는 병원에서 성폭력 [성희롱(말), 성추행(행동), 성폭행]을 당한 적이 있습니까?

1. 예 2. 아니오

25. 성폭력 [성희롱(말), 성추행(행동), 성폭행]의 가해자가 누구 였습니까? (복수응답 가능) (24번 “예”라고 답하신 분만 응답해주세요)

1. 교수 2. 펠로우 3. 상급 전공의 4. 환자에게 5. 그 외(동료 또는 직원 등, 환자 제외)에게

26. 귀하는 병원에서 언어적 폭력을 당한 적이 있습니까?

1. 예 2. 아니오

27. 언어적 폭력의 가해자가 누구 였습니까? (복수응답 가능) (26번 “예”라고 답하신 분만 응답해주세요)

1. 교수 2. 펠로우 3. 상급 전공의 4. 환자에게 5. 그 외(동료 또는 직원 등, 환자 제외)에게

28. 귀하는 병원에서 신체적 폭력을 당한 적이 있습니까?

1. 예 2. 아니오

29. 신체적 폭력의 가해자가 누구 였습니까? (복수응답 가능) (**28**번 “예”라고 답하신 분만 응답해주세요)

1. 교수 2. 펠로우 3. 상급 전공의 4. 환자에게 5. 그 외(동료 또는 직원 등, 환자 제외)에게

30. 귀하는 교수 또는 지도전문의로부터 논문을 이용한 협박(모든 형태)을 당한 적이 있습니까?

1. 예 2. 아니오

31. 병원내 폭력 사건(언어, 신체폭력 모두 포함) 발생시 병원내의 처리절차가 확립되어있습니까?

1. 예 2. 아니오 3. 모른다

32. 병원내 폭력 사건(언어, 신체폭력 모두 포함) 발생시 피해자 보호가 잘 이루어지고 있습니까?

1. 예 2. 아니오 3. 모른다

33. 병원내 성폭력 사건(성희롱, 성추행, 성폭행 모두 포함) 발생시 병원내의 처리절차가 확립되어있습니까?

1. 예 2. 아니오 3. 모른다

34. 병원내 성폭력 사건(성희롱, 성추행, 성폭행 모두 포함) 발생시 피해자 보호가 잘 이루어지고 있습니까?

1. 예 2. 아니오 3. 모른다

**35.< 전공의 수련 환경 주관적 평가1>**

|  | ◀그렇지 않다. 그렇다.▶  ① ② ③ ④ ⑤ |
| --- | --- |
| -1. 전반적인 근무환경에 만족한다. | ○ ○ ○ ○ ○ |
| -2. 근무 강도에 만족한다. | ○ ○ ○ ○ ○ |
| -3. 근무시간(일평균)이 적절하다. | ○ ○ ○ ○ ○ |
| -4. 급여 지급 수준에 만족한다. | ○ ○ ○ ○ ○ |
| -5. 당직수당 및 기타 상여금 지급 수준에 만족한다. | ○ ○ ○ ○ ○ |
| -6. 타직원과의 처우에 대한 평등성이 적절하다. | ○ ○ ○ ○ ○ |
| -7. 근로 환경(당직실 포함) 및 전반적인 시설에 만족한다. | ○ ○ ○ ○ ○ |
| -8. 병원 직원 식당 청결도 및 맛에 만족한다. | ○ ○ ○ ○ ○ |
| -9. 기타 전공의 복지 환경에 만족한다. | ○ ○ ○ ○ ○ |
| -10. 의료인 간의 뚜렷한 업무 분배가 되어 있다. | ○ ○ ○ ○ ○ |
| -11. 병원 내 사회생활에 있어, 상호간 인격 존중을 한다. | ○ ○ ○ ○ ○ |
| -12. 삶의 질이 높다. | ○ ○ ○ ○ ○ |
| -13. 휴가(연가, 병가, 출산휴가 포함) 사용이 자유롭다. | ○ ○ ○ ○ ○ |
| -14. 결혼, 임신, 경조사 등 개인적인 사정에 대한 스케줄의 유연성이 있다. | ○ ○ ○ ○ ○ |
| -15. 병원내 폭력(언어, 신체폭력 포함) 사건 발생시 병원내 처리절차를 신뢰할 수 있다. | ○ ○ ○ ○ ○ |
| -16. 병원내 성폭력 사건(희롱, 추행 포함) 사건 발생시 병원내 처리절차를 신뢰할 수 있다. | ○ ○ ○ ○ ○ |
| -17. 전반적인 교육환경에 만족한다. | ○ ○ ○ ○ ○ |
| -18. 환자군과 질환의 다양성이 있다. | ○ ○ ○ ○ ○ |
| -19. 임상능력 함양을 위한 참여 기회(시술 참여, 의사결정과정 참여 등)가 있다. | ○ ○ ○ ○ ○ |
| -20. 병동, 외래, 응급실, 중환자실, 수술실 등의 수련과정이 고르게 분포하고 있다. | ○ ○ ○ ○ ○ |
| -21. 지도전문의에게 적절한 교육적 지도를 받고 있다. | ○ ○ ○ ○ ○ |
| -22. 수련과정에 학술활동(집담회 등)이 충분히 포함되어 있고 효과적이다 | ○ ○ ○ ○ ○ |
| -23. 교육자료(학술논문, 인터넷 자료 등) 접근도에 만족한다. | ○ ○ ○ ○ ○ |
| -24. 해외 연수 및 학회 참여 기회에 만족한다. | ○ ○ ○ ○ ○ |
| -25. 수련과정을 통해 전문적 의학지식이 함양된다. | ○ ○ ○ ○ ○ |
| -26. 수련과정을 통해 의료진과 환자간의 의사소통 능력이 향상된다. | ○ ○ ○ ○ ○ |
| -27. 현재 수련과정 후 결과적으로 환자진료 능력이 향상된다. | ○ ○ ○ ○ ○ |

**36< 전공의 수련 환경 주관적 평가2>**

**인턴 제외하고 레지던트만 답해주세요 -> 필수응답 아님**

| -1. 타 수련병원에의 파견 수련에 만족한다. | ○ ○ ○ ○ ○ |
| --- | --- |
| -2. 해외 연수 및 학회 참여 기회에 만족한다. | ○ ○ ○ ○ ○ |
| -3. 연차별(인턴 포함)로 수련 교육목표가 제시되어 있다. | ○ ○ ○ ○ ○ |
| -4. 연차별 수련과정을 통해 수련의 실제 교육목표를 달성할수 있다. | ○ ○ ○ ○ ○ |
| -5. 연차별 수련과정을 통해 전문적 의학 술기 능력이 향상된다. | ○ ○ ○ ○ ○ |
| -6. 연차별 수련과정의 구분이 확실하다. | ○ ○ ○ ○ ○ |
| -7. 연차별 수련이 체계적으로 진행되고 있다. | ○ ○ ○ ○ ○ |
| -8. 수련의 양이 연차별로 고르게 분포되어 있다. | ○ ○ ○ ○ ○ |
| -9. 현재 전공의 수련이 수련 직후 봉직을 하거나 개원하는데 충분하다. | ○ ○ ○ ○ ○ |
| -10. 현 수련내용이 충분하여 별도 전임의 과정이 불필요하다. | ○ ○ ○ ○ ○ |

**<환자 안전>**

37. 정규 근무 시 주치의를 맡은 경우 입원 환자는 평균 몇 명 담당합니까?

1. 해당 사항 없음 2. 약 ( ) 명

38. 당직 근무 시 주치의를 맡은 경우 환자를 평균 몇 명 담당합니까?

1. 해당 사항 없음 2. 약 ( ) 명

39. 일반 진료 시, 외래환자를 1시간당 평균 몇명 진료합니까?

1. 해당 사항 없음 2. 약 ( ) 명/시간

40. 교수의 외래 진료를 도울 시, 교수는 외래환자를 1시간당 평균 몇명 진료합니까?

1. 해당 사항 없음 2. 약 ( ) 명/시간

41. 정규 수술은 하루 평균 몇 시간 입니까? (외과 계열과 마취과 전공의만 답해주세요)

1. 약 ( ) 시간

**-> 필수응답 아님**

42. 당직 수술은 하루 평균 몇 시간 입니까? (외과 계열과 마취과 전공의만 답해주세요)

1. 약 ( ) 시간

**-> 필수응답 아님**

43. 하루 평균 응급실 내원 환자는 몇 명 입니까? (응급의학과 전공의만 답해주세요)

**1.** 약 ( ) 명

**-> 필수응답 아님**

44. 하루 평균 응급실 내 의사 인력은 몇 명 입니까? (응급의학과 전공의만 답해주세요)

**1.** 약 ( ) 명

**-> 필수응답 아님**

45. 누적된 피로와 불충분한 수면으로 환자에게 올바른 진료를 시행하지 못한 적이 있습니까?

1. 예 2. 아니오

46. 과도한 업무량으로 인해 환자에게 올바른 진료를 시행하지 못한 적이 있습니까?

1. 예 2. 아니오

47. 환자가 귀하에게 가한 신체적/언어적 폭력으로 인해 환자에게 올바른 진료를 시행하지 못한 적이 있습니까?

1. 예 2. 아니오

48. 의학지식의 부족으로 인해 환자에게 올바른 진료를 시행하지 못한 적이 있습니까?

1. 예 2. 아니오

49. 상급자의 능력부족으로 인해 환자에게 올바른 진료를 시행하지 못한 적이 있습니까?

1. 예 2. 아니오

50. 귀하가 당직 근무 시 응급환자에 대해 담당교수와 논의하려고 할 때 신속하고 원활하게 소통 했다고 생각하는 비율은 몇 % 입니까?

( ) %

51. 귀하의 병원에 무면허진료보조인력 (UA, Unlicensed Assistant)가 있습니까?

* UA(Unlicensed Assistant)는 합법적이지 않으나, 의사의 업무 중 일부를 위임받아 대신하는 간호사를 말하며, Physician Assistant (PA), Surgery Assistant, Nurse Practitioner 등 부르는 명칭이 다양하다.

1. 예 2. 아니오

52. 귀하의 병원에서 무면허진료보조인력 (UA, Unlicensed Assistant)가 독립적으로 침습적 술기를 직접 하는 것을 본 적이 있습니까? (침습적 술기: 동맥천자, A line, 중심정맥관 삽입, 뇌척수액 검사, 흉관 삽입, 복수천자) (51번 “예”라고 답하신 분만 응답해주세요)

1. 예 2. 아니오

53. 귀하의 병원에서 무면허진료보조인력 (UA, Unlicensed Assistant)가 독립적으로 약 처방 하는 것을 본 적이 있습니까? (51번 “예”라고 답하신 분만 응답해주세요)

1. 예 2. 아니오

54. 귀하의 병원에서 무면허진료보조인력 (UA, Unlicensed Assistant)가 수술을 직접 집도하는 것을 본 적이 있습니까? (51번 “예”라고 답하신 분만 응답해주세요)

1. 예 2. 아니오

55. 귀하께서 무면허진료보조인력 (UA, Unlicensed Assistant)로 인해 교육적 기회를 박탈당했다고 느낀 적이 있습니까? (51번 “예”라고 답하신 분만 응답해주세요)

1. 예 2. 아니오

**설문에 응해주셔서 대단히 감사합니다.**

**2018 전국 전공의 병원평가 설문조사 (문항 개발)**

1. **개인 정보**

Q1. 귀하의 성별을 다음의 보기에서 선택해 주시기 바랍니다.

1) 남성 2) 여성

Q2. 귀하의 만 나이를 아래에 기입해 주시기 바랍니다.

만_____세

Q3. 귀하의 수련병원은? (설문조사는 익명으로 진행됩니다)

_________________ (주관식 서술)

Q4. 귀하의 수련 연차를 다음의 보기에서 선택해 주시기 바랍니다.

1) 인턴 2) 레지던트 1년 3) 레지던트 2년 4) 레지던트 3년 5) 레지던트 4년

Q5. 귀하의 수련 과를 다음의 보기에서 선택해 주시기 바랍니다.

1) 인턴 2) 가정의학과 3) 결핵과 4) 내과 5) 마취통증의학과
6) 방사선종양학과 7) 병리과 8) 비뇨의학과 9) 산부인과 10) 성형외과 11) 소아청소년과

12) 신경과 13) 신경외과 14) 안과 15) 영상의학과 16) 외과

17) 응급의학과 18) 이비인후과 19) 재활의학과 20) 정신건강의학과

21) 정형외과 22) 직업환경의학과 23) 진단검사의학과 24) 피부과

25) 핵의학과 26) 흉부외과 27) 예방의학과 28) 기타 ( )

Q6. 귀하의 주관적 건강 수준을 다음의 보기에서 선택해 주시기 바랍니다. (또래와 비교했을 때)

1) 아주 건강하다

2) 건강한 편이다

3) 보통이다

4) 가끔 이상이 있다

5) 자주 이상이 있다

Q7. 귀하의 주관적 삶의 질을 다음의 보기에서 선택해 주시기 바랍니다.

1) 아주 좋다

2) 좋다

3) 보통이다

4) 나쁘다

5) 아주 나쁘다

Q8. 경품 추첨을 위해 본인의 연락처(핸드폰 번호)를 적어주십시오. (필수응답 아님)

(010-0000-0000)

_____________________________________

**B. 전공의 근무환경**

**(근무시간, 휴식시간, 급여, 시설 및 복지, 인간관계)**

**<근무 환경 전반>**
Q1. 귀하의 전반적인 근무환경에 만족하십니까?

1) 매우 만족 2) 만족 3) 보통 4) 불만족 5) 매우 불만족

Q2. (Q1.에서 4, 5 선택한 경우) 근무환경에 만족하지 못하는 이유는 무엇입니까? (다중 선택)

1) 근무 시간 2) 근무 강도 3) 급여 4) 시설 5) 복지 6) 인간 관계 7) 기타 (주관식 서술)

Q3. 2015.12 제정된 [전공의의 수련환경 개선 및 지위 향상을 위한 법률] (이하 전공의법)에서는 전공의의 근무 시간에 대한 사항을 다음과 같이 규정하고 있습니다.

1. *주당 근무시간 80시간 제한(교육적 목적으로 8시간까지 연장 가능)*
2. *36시간이 초과하는 연속근무 금지(응급 상황인 경우 4시간까지 연장 가능)*
3. *근무와 다음 근무 사이에 최소 10시간의 휴식시간 부여*

** 참고: 전공의법 전문 http://likms.assembly.go.kr/law/lawsLawtInqyDetl1010.do?mappingId=%2FlawsLawtInqyDetl1010.do&genActiontypeCd=2ACT1010&genDoctreattypeCd=DOCT2004&contId=2015122200000005&contSid=0001&cachePreid=ALL&genMenuId=menu_serv_nlaw_lawt_1010&viewGb=PROM)*

Q4. 귀하가 근무하는 병원에서는 전공의법에 명시된 바가 잘 지켜지고 있습니까?

1) 철저히 지켜진다 2) 어느정도 지켜진다 3) 보통이다 4) 잘 지켜지지 않는다 5) 전혀 지켜지지 않는다

**<근무 시간>**

Q5. 최근 6개월 간, **평균** 1주일에 몇 시간 근무하십니까?
(근무시간은 병원에 있었던 총 시간으로 계산하며, 1주일은 총 168시간입니다.)

____ 시간 / 1주일

Q6. 최근 6개월 간, **최대** 1주일에 몇 시간 근무하셨습니까?

(근무시간은 병원에 있었던 총 시간으로 계산하며, 1주일은 총 168시간입니다.)

____ 시간 / 1주일

Q7. 귀하는 지난 4주 동안 최대 연속 수련시간인 36시간을 초과한 적이 한 번 이상 있습니까?

1) 예 2) 아니오

Q8. (Q7.에서 1을 선택한 경우) 당시 연속해서 몇 시간 근무하셨습니까?

______ 시간

Q9. 1주일에 야간 당직근무(응급의학과의 경우 야간 근로)를 평균 몇 회 하십니까 ?

____ 회 / 1주일

Q10. 귀하가 지금까지 경험한 최대 연속 당직일수는 며칠입니까?

______ 일

**<휴식 시간>**

Q11. 당직 근무 종료 후 정규시작 전까지 실질적인 휴식시간은 몇 시간 입니까?

1) ______ 시간 2) 해당 없음 (당직 근무가 없는 과의 경우 해당 없음에 표시)

Q12. 근무 중 귀하의 하루 평균 수면시간은 몇 시간 입니까?

____ 시간 / 1일

Q13. 점심 식사시간에 식사를 할 수 있는 경우가 일주일에 몇 번이나 되십니까?

____ 번 / 1주일

Q14. 한 끼 식사 시에 사용할 수 있는 실질적인 시간(식사시간 + 휴식시간)은 얼마나 되십니까?

______ 분

Q15. 식사 시간에 받는 콜은 평균 몇 번 정도 입니까?

______ 번

Q16. 귀하는 근무 스케줄 상 오프임에도 불구하고 근무를 지속해야 했던 적이 있습니까?
(월급 산정 시 근무 시간으로 계산되지 않는 시간 외 근무)

1) 예 2) 아니오

Q17. (Q16.에서 1 선택한 경우) 오프임에도 근무를 지속 해야 했던 이유는 무엇입니까? (다중 응답)

1) 회진 2) 외래 3) 수술 4) 주치의 업무 5) 간호사 콜 6) 응급 상황 7) 교육 8) 연구 및 논문

9) 과내 행사 (회식, 학회) 10) 잡일 (서류작업 등) 11) 기타 (주관식 서술)

Q18. (Q16.에서 1 선택한 경우) 위와 같은 사유로 근무를 지속하게 되는 시간은 일주일에 총 합하여 평균 몇 시간 입니까?

______ 시간 / 1주일

Q19. 다른 병원으로 파견근무를 할 경우 (예: 서울아산병원 → 홍천아산병원), 병원 이동시 이동, 휴식, 업무 인계에 필요한 충분한 시간을 보장하고 있습니까?

1) 예 2) 아니오 3) 파견 근무 없음

Q20. 1개월 근무 시 휴일(24시간 출근하지 않는 날, 휴가 제외)은 며칠이나 부여 받습니까?

*※ 휴일(off): 연차휴가(14일)를 제외하고 24시간 연속으로 쉬는 날, 토일, 공휴일 포함*

*※ 휴일은 완전한 Duty-off로, house-call을 받고 나간 경우는 휴일에서 제외*

____ 일 / 1개월

Q21. 1년간 부여 받은 휴가는 총 며칠 입니까? (주말 및 공휴일 제외)

____ 일 / 1년

Q22. (2018년 인턴, 레지던트로 근무 당시) 부여 받은 휴가는 실제로 보장 되었습니까?

1) 예 2) 아니오 3) 해당사항 없음

**<급여>**

Q23. 귀하의 근로 계약서에 급여 관련 사항이 명시되어 있었습니까?

1) 그렇다 2) 잘 모르겠다 3) 아니다

Q24. 최근 6개월 간, 귀하의 한달 급여는 평균 얼마입니까? (세후 기준)

______ 만원

Q25. 최근 6개월 간, 귀하의 평일 하루 평균 야간 당직비는 얼마입니까 ? (일단위로 환산하여 작성)

1) 평일 ______ 만원 / 일 2) 해당 사항 없음 3) 모르겠다

Q26. 최근 6개월 간, 귀하의 휴일 하루 평균 야간 당직비는 얼마입니까 ? (일단위로 환산하여 작성)

1) 휴일 ______ 만원 / 일 2) 해당 사항 없음 3) 모르겠다

Q27. 최근 6개월 간, 실제 당직 근무를 했음에도 당직비 지급을 받지 못한 경우가 있습니까?
 (콜당직, 백당직 포함)

1) 있다 2) 없다 3) 모르겠다

Q28. (Q27.에서 1을 선택한 경우) 지급받지 못한 당직비를 환산하면 월 평균 얼마 정도입니까?

평균 ______ 만원 / 월

**<시설 및 복지>**

Q29. 귀하가 근무하는 병원 내 전반적인 근로 시설에 만족하십니까?

1) 예 2) 아니오

Q30. (Q.29에서 2를 선택한 경우) 만족하지 못하는 이유는 무엇입니까? (다중 응답)

1) 시설 부재 2) 공간 협소 3) 필요 물품 부족 (PC, 프린터 등) 4) 청소 등 관리 소홀 5) 기타 (주관식)

Q31. 귀하가 근무하는 병원에서는 전공의를 위한 충분한 휴게공간 (당직실 포함)이 제공되십니까?

1) 예 2) 아니오

Q32. 귀하가 근무하는 병원은 전공의 복지와 관련해 담당부서 (교육수련부 등)와 소통이 잘 이루어집니까?
1) 예 2) 아니오

Q33. 귀하가 근무하는 병원은 전공의를 위한 대표 단체 (전공의협의회 등)가 존재합니까?

1) 예 2) 아니오

Q34. 귀하가 근무하는 병원에서는 전공의 복지 지원을 위해 따로 지급하는 예산이 있습니까? (병원에서 자체적으로 편성하여 지급하는 예산 만을 의미)

1) 예 2) 아니오 3) 모르겠다.

Q35. (Q34.에서 1을 선택한 경우) 예산은 1년간 얼마나 지급 됩니까? (개인당 배당 되는 금액으로 환산)

________ 원 / 개인, 1년간

Q36. 귀하가 근무하는 병원에서는 휴가(연가, 병가, 출산휴가 포함) 사용이 자유롭나요?

1) 예 2) 아니오

Q37. 결혼, 임신, 경조사 등 개인적인 사정에 대한 스케줄의 유연성이 있습니까?

1) 예 2) 아니오

Q38. (Q36, Q37 중 하나라도 2를 선택한 경우) 그 이유는 무엇입니까?

1) 행정 처리 문제 2) 과내 분위기 및 방침 3) 근무 조율 불가 4) 상급자의 눈치 5) 동료에 대한 미안함
6) 차후 불이익 우려 7) 기타 (주관식 서술)

**※ 전공의법 제 8조에서는 여성전공의의 출산 관련 휴가에 대해서 「근로기준법」 을 준용토록 하고 있으며, 추가 수련에 관한 사항을 보건복지부 산하의 수련환경평가위원회에서 정하도록 하고 있습니다. 이에 따르면 여성 전공의가 임신을 하는 경우 임신을 인지하는 시점부터 산후 1년까지 출산 휴가 3개월을 제외한 기간 동안 야간근무를 포함 주 40시간이 넘는 노동은 원칙적으로 금지되어 있습니다**.

*** 참고 : 전공의법**

***제8조*** *① 여성전공의에 대한 출산전후휴가 및 유산·사산 휴가에 관하여는 근로기준법 제74조제1항부터 제4항까지를 따른다. (http://likms.assembly.go.kr/law/lawsLawtInqyPopu1070.do)*

*② 제1항에 따라 발생하는 추가 수련에 관한 사항은 제15조에 따른 수련환경평가위원회에서 정하는 바에 따른다.*

**** 참고 : 근로기준법***

***제70조제2항*** *사용자는 임산부와 18세 미만자를 오후 10시부터 오전 6시까지의 시간 및 휴일에 근로시키지 못한다. 다만, 다음 각 호의 어느 하나에 해당하는 경우로서 고용노동부장관의 인가를 받으면 그러하지 아니하다.*

*- 산후 1년이 지나지 아니한 여성의 동의가 있는 경우*

*- 임신 중의 여성이 명시적으로 청구하는 경우*

***제71조*** *사용자는 산후 1년이 지나지 아니한 여성에 대해서는 단체협약이 있는 경우라도 1일에 2시간, 1주일에 6시간, 1년에 150시간을 초과하는 시간외근로를 시키지 못한다.*

***제74조 ①*** *사용자는 임신 중의 여성에게 90일(한 번에 둘 이상 자녀를 임신한 경우에는 120일)의 출산전후휴가를 주어야 한다. .. ⑦ 임신 후 12주 이내 또는 36주 이후에 있는 여성 근로자가 1일 2시간의 근로시간 단축을 신청하는 경우 이를 허용하여야 한다.*

Q39. 귀하는 지금까지의 전공의 과정 중 임신 및 출산 경험이 있습니까?

1) 예 2) 아니오

Q40. (Q.39에서 1을 선택한 경우) 임신 및 출산 과정에서 힘들었던 점은 무엇이 있습니까?

1) 과중한 업무에 따른 신체적 부담 2) 출산 휴가 등에 따른 과내 눈초리 3) 진로/선발에 따른 불이익

4) 업무 분담에 따른 미안함 5) 수련 기간 감소에 따른 아쉬움 6) 기타 (주관식 서술)

Q41. 일부 학회에서는 임신 전공의의 근무시간 감소에 따라 전문의 자격 취득을 위한 추가수련이 필요하다고 주장하고 있습니다. 이에 찬성하십니까?

1) 찬성 (추가 수련이 필요하다) 2) 반대 (추가 수련이 필요하지 않다)

Q42. 전공의의 진정한 모성보호를 이룩하기 위해 보완할 점이 있다면 기술해 주십시오.

______________________________________ (주관식 서술)

**<인간 관계>**

Q43. 귀하는 근무 중 타인과 관계로부터 스트레스를 받은 적이 있습니까?

1) 자주 있다 2) 가끔 있다 3) 거의 없다 4) 전혀 없다

Q44. 스트레스의 정도를 점수로 표현하면 몇 점인가요?
(사직을 고려할 정도로 매우 극심한 경우 10, 전혀 없는 경우 0)

________ 점

Q45. (Q43.에서 1,2를 선택한 경우) 주로 어느 직종과 문제를 겪습니까? (다중 선택)

1) 교수 2) 펠로우 3) 동일과 상급 전공의 4) 동일과 후배 전공의 5) 동기 6) 타과 전공의 7) 간호사
8) 행정직원 9) 의료기사 10) 환자 및 보호자 11) 기타 (주관식 서술)

Q46. (Q43.에서 1,2를 선택한 경우) 주로 어떤 문제로 인해 스트레스를 받습니까?

1) 과도한 훈육 2) 일방적인 괴롭힘 (태움) 3) 업무 충돌 4) 사적 문제 5) 기타 (주관식 서술)

**C. 전공의 수련 환경**

**<전공의 선발>**

Q1. 전공의(인턴, 레지던트) 선발과정이 공정하고 객관적으로 이루어진다고 생각하십니까?

1) 예 2) 아니오

**<전공의 직무 간 훈련>**

Q2. 전공의가 수행해야 할 업무를 상세히 기술한 문서(직무기술서)가 있습니까?

1) 예 2) 아니오

Q3. 직무기술서에는 수행된 업무의 수준을 평가하는 역량 기준이 기술되어 있습니까?

1) 예 2) 아니오

Q4. 전공의의 현재 역량과 주어진 업무에 필요한 역량이 얼마나 차이나는지 확인하고 있습니까?

1) 예 2) 아니오

Q5. 전공의가 해당 업무를 통해 배우기로 되어 있는 지식, 기술, 태도를 갖추는데 필요한 학습 과정이 적절하게 구성되어 있습니까?

1) 매우 그렇다

2) 그렇다

3) 보통이다

4) 아니다

5) 전혀 아니다

Q6. 교수, 전임의, 상급 전공의 등은 학습 과정에 효과적으로 기여하고 있습니까?

1) 매우 그렇다

2) 그렇다

3) 보통이다

4) 아니다

5) 전혀 아니다

Q7. 업무에 필요한 역량은 주로 어떤 경로를 통해 배우고 있습니까?

1) 교수 2) 펠로우 3) 상급 전공의 4) 동료 전공의 5) 독학 6) 기타 (주관식 서술)

Q8. 수련교과과정에 제시된 임상경험을 달성하는 데 필요한 업무량을 100이라고 봤을 때 실제로 수행하고 있는 업무량은 몇 정도입니까?

( )

Q9. 전체 업무 중 수련과 관련 없는 업무가 차지하는 비중이 얼마나 됩니까? (예시 : 의국 내 서류정리 등과 같이 행정직원이 대체 가능한 업무 또는 본인과 관련 없는 논문잡일 등)

( ) %

Q10. 수행한 업무의 결과에 대해 적절한 시간에, 적절한 빈도로 정확한 피드백을 받습니까?

1) 예 2) 아니오

Q11. 전공의가 업무를 수행한 후 역량이 얼마나 향상되었는지 적절하게 평가하고 있습니까?

1) 예 2) 아니오

**<전공의 술기 교육>**

Q12. (공통) 최근 1년 간 술기 교육을 받은 후 적절한 시간에, 적절한 빈도로 직접 수행해 볼 기회를 받은 적이 있습니까?

1) 예 2) 아니오

Q13. 최근 1년 간 술기 교육을 받은 후 2주 이내에 술기의 일부 혹은 전체를 수행해 볼 기회를 얻은 것이 몇 번입니까?

______회

Q14. (술기 수행이 이루어진 경우) 술기 수행은 교수나 전임의의 적절한 지도감독 아래에 이루어졌습니까?

1) 예 2) 아니오

Q15. (외과계) 최근 1달 간 수술에 참여한 횟수는 몇 번입니까?

______회

Q16. (외과계) 최근 1달 간 1^st^ assistant로 수술에 참여한 횟수는 몇 번입니까?

______회

Q17. (외과계) 최근 1달 간 집도의로 수술에 참여한 횟수는 몇 번입니까?

______회

**<수련불평등>**

Q18. 수련병원등은 수련 프로그램의 질을 높이기 위해 시간, 인력, 시설, 재원이 얼마나 필요한지 검토하고 있습니까?

1) 예 2) 아니오

Q19. 수련병원등은 수련 프로그램의 질을 높이기 위해 필요한 시간, 인력, 시설, 재원에 해당하는 비용을 지불하고 있습니까?

1) 예 2) 아니오

Q20. (레지던트) 레지던트를 시작한 이후 국내 및 국제 학회에 각각 몇 번 참석하였습니까?

1) 국내 학회 : ( ) 회, 국제 학회 : ( ) 회

2) 둘다 참석한 적 없음

Q21. (레지던트) 레지던트를 시작한 이후 국제 학회에 몇 번 참석하였습니까?

( ) 회

Q22. (레지던트) 학회 참석 시 소속 과 또는 병원이 부담한 비용은 전체 비용의 몇 %입니까? (참석하지 못한 경우 결측치 처리)

( ) %

Q23. 최근 2달 간 경험한 전공의 수련 프로그램에 얼마나 만족하십니까?

1) 매우 만족한다

2) 만족한다

3) 보통이다

4) 만족하지 않는다

5) 전혀 만족하지 않는다

**<전공의 수련의 완결성>**

Q24. 전공의 수련 프로그램을 성실히 마치고 나면 전문의로서 단독으로 일하는데 역량이 충분할 것이라고 생각하십니까?

1) 매우 그렇다

2) 그렇다

3) 보통이다

4) 아니다

5) 전혀 아니다

Q25. 전임의 과정에 들어가지 않으면 전문의로서 단독으로 일하는데 필요한 역량을 배울 수 없다고 생각하십니까?

1) 매우 그렇다

2) 그렇다

3) 보통이다

4) 아니다

5) 전혀 아니다

**D. 전공의 안전**

**<병원 내 폭력>**

Q1.병원에서 언어적 폭력을 당한 적이 있습니까?

1) 예 2) 아니오

Q2. (Q1.에서 1을 선택한 경우) 언어적 폭력의 가해자가 누구였습니까? (복수응답 가능)

1) 교수 2) 펠로우 3) 상급 전공의 4) 환자 5) 그 외(동료 또는 직원 등, 환자 제외)에게

Q3. 병원에서 신체적 폭력을 당한 적이 있습니까?

1) 예 2) 아니오

Q4. (Q3.에서 1을 선택한 경우) 신체적 폭력의 가해자가 누구 였습니까? (복수응답 가능)

1) 교수 2) 펠로우 3) 상급 전공의 4) 환자 5) 그 외(동료 또는 직원 등, 환자 제외)에게

Q5. 교수 또는 지도전문의로부터 논문 혹은 진로를 빌미로 협박을 당한 적이 있습니까?

1) 예 2) 아니오

Q6. 병원내 폭력 사건(언어, 신체폭력 모두 포함) 발생시 병원내의 처리절차가 확립되어있습니까?

1) 예 2) 아니오 3) 모른다

Q7. 병원내 폭력(언어, 신체폭력 포함) 사건 발생시 병원내 처리절차를 신뢰하십니까?

1) 예 2) 아니오

Q8. 병원내 폭력 사건(언어, 신체폭력 모두 포함) 발생시 피해자 보호가 잘 이루어지고 있습니까?

1) 예 2) 아니오 3) 모른다

**<병원 내 성폭력>**

Q9. 귀하는 병원에서 성폭력 [성희롱(언어), 성추행(행동), 성폭행]을 당한 적이 있습니까?

1) 예 2) 아니오

Q10. (Q9.에서 1을 선택한 경우) 성폭력 [성희롱(말), 성추행(행동), 성폭행]의 가해자가 누구 였습니까? (복수응답 가능)

1) 교수 2) 펠로우 3) 상급 전공의 4) 환자 5) 그 외(동료 또는 직원 등, 환자 제외)에게

Q11. 병원내 성폭력 사건(성희롱, 성추행, 성폭행 모두 포함) 발생시 병원내의 처리절차가 확립되어있습니까?

1) 예 2) 아니오 3) 모른다

Q12. 병원내 성폭력(성희롱, 성추행 포함) 사건 발생시 병원내 처리절차를 신뢰하십니까?

1) 예 2) 아니오

Q13. 병원내 성폭력 사건(성희롱, 성추행, 성폭행 모두 포함) 발생시 피해자 보호가 잘 이루어지고 있습니까?

1) 예 2) 아니오 3) 모른다

**<환자에 의한 폭력>**

Q14. 환자가 가한 언어적 폭력으로 인해 진료 수행에 방해받은 적이 있습니까?

1) 예 2) 아니오

Q15. (있다면) 최근 6개월 간 몇 번 정도 환자의 언어적 폭력으로 인해 진료 수행에 방해를 받았습니까?

( ) 회

Q16. 환자가 가한 신체적 폭력으로 인해 진료 수행에 방해받은 적이 있습니까?

1) 예 2) 아니오

Q17. (있다면) 최근 6개월 간 몇 번 정도 환자의 신체적 폭력으로 인해 진료 수행에 방해를 받았습니까?

( ) 회

Q18. 환자가 가한 신체적 폭력으로 인해 근무에 바로 복귀하지 못할 정도의 상해를 입은 적이 있습니까?

1) 예 2) 아니오

**<방사능 노출, 감염 노출>**

*대한전공의협의회에서 수행한 방사선 노출량 측정 조사에 따르면 조사에 참여한 전공의들은 표층 선량 기준으로 적게는 0.01mSv에서 많게는 9.52mSv까지 노출되고 있었습니다(이는 비교적 피폭량이 많은 흉부CT 촬영 시 노출되는 것과 비슷합니다).*

Q19. 하루에 한 번 이상 방사능에 노출되는 업무를 수행하고 계십니까?

1) 예 2) 아니오

Q20. (“예”라고 응답한 경우) 병원이 방사능 노출로 인한 위험을 방지하기 위해 적절한 조치(보호구 제공, 계수기 부착, 노출량 관리 등)를 취하고 있습니까?

1) 예 2) 아니오

Q21. 병원이 감염원에 노출되는 상황으로 인한 위험을 방지하기 위해 적절한 조치(보호구 제공, 노출 시 사후 조치 등)를 취하고 있습니까?

1) 예 2) 아니오

**E. 환자 안전**

**(환자 수 및 업무 로딩, 무면허진료보조행위, 감염관리)**

Q1. 귀하의 가족이 아플 때, 본인이 수련 중인 병원으로 모시고 올 의향이 있습니까?
(환자 안전 및 수준 높은 치료 측면에서)

1) 모시고 올 것이다 2) 고려해 볼 것이다 3) 절대 모시고 오지 않을 것이다.

Q2. (Q1.에서 3을 선택한 경우) 구체적인 이유가 있다면 기술해 주시기 바랍니다.

________________________ (주관식 서술)

**<환자 수>**

Q3. **정규 근무** 시 주치의를 맡은 경우 입원 환자를 **평균** 몇 명 담당합니까?

1) 평균 _____ 명 2) 해당 사항 없음

Q4. **당직 근무** 시 **최대** 몇 명의 환자를 담당합니까?

1) 최대 _____ 명 2) 해당 사항 없음

Q5. **당직 근무** 시 과 내 의사 인력은 **평균** 몇 명 입니까?

1) 평균 _____ 명 2) 해당 사항 없음

Q6. (외과 계열 및 마취과 전공의 대상) 하루 수술에 참여하는 시간은 **평균** 몇 시간입니까?
1) 약 _____ 시간 / 1일 2) 해당 사항 없음

Q7. (외과 계열 및 마취과 전공의 대상) 하루 수술에 참여하는 시간은 **최대** 몇 시간입니까?
1) 약 _____ 시간 / 1일 2) 해당 사항 없음

Q8. (응급의학과 전공의 대상) 하루 평균 응급실 내원 환자는 몇 명 입니까?

1) 약 _____ 명 / 1일 2) 해당 사항 없음

Q9. 근무 중 환자에게 적절한 의학적 처치가 불가 했던 적이 있었습니까?

1) 예 2) 아니오

Q10. (Q9.에서 1을 선택한 경우) 적절한 처치가 불가능 했던 원인 무엇이었습니까? (다중 선택)

1) 과도한 환자수 2) 과중한 업무량 3) 응급상황 동시 발생 4) 입원실/중환자실/수술방 부족
5) 인력 부족 (의사, 간호사) 6) 병원 내 인프라 한계 (검사, 처치 등) 7) 노티 및 의사결정 단계 지연

8) 타과와 협력 문제 9) 환자/보호자 소통 문제 10) 기타 (주관식 서술)

**< 무면허진료보조인력 >**

Q11. 귀하의 병원에 무면허진료보조인력 (UA, Unlicensed Assistant)가 있습니까?

* UA(Unlicensed Assistant)는 합법적이지 않으나, 의사의 업무 중 일부를 위임받아 대신하는 간호사를 말하며, Physician Assistant (PA), Surgery Assistant, Nurse Practitioner 등 부르는 명칭이 다양하다.

1) 예 2) 아니오

Q12. (Q11.에서 1을 선택한 경우) 귀하의 병원에서 무면허진료보조인력 (UA, Unlicensed Assistant)가 독립적으로 침습적 술기를 직접 하는 것을 본 적이 있습니까?
(침습적 술기: 동맥천자, A line, 중심정맥관 삽입, 뇌척수액 검사, 흉관 삽입, 복수천자)

1) 예 2) 아니오

Q13. (Q11.에서 1을 선택한 경우) 귀하의 병원에서 무면허진료보조인력 (UA, Unlicensed Assistant)가 독립적으로 약 처방 하는 것을 본 적이 있습니까?

1) 예 2) 아니오

Q14, (Q11.에서 1을 선택한 경우) 귀하의 병원에서 무면허진료보조인력 (UA, Unlicensed Assistant)가 수술을 직접 집도하는 것을 본 적이 있습니까?

1) 예 2) 아니오

Q15, (Q11.에서 1을 선택한 경우) 귀하께서 무면허진료보조인력 (UA, Unlicensed Assistant)로 인해 교육적 기회를 박탈당했다고 느낀 적이 있습니까?
1) 예 2) 아니오

**<보건의료체계 >**

Q16. 보험 수가 불인정 등 비용 혹은 병원 방침 문제로 인해 환자에게 최선의 진료를 하지 못했던 적이 있습니까?

1) 예 2) 아니오

Q17. (Q16.에서 1을 선택한 경우) 의학적으로 필요 함에도 환자에게 최선의 진료가 불가했다면, 이에 해당하는 항목을 선택 혹은 기술해주십시오.

1) 영상 검사 2) 혈액 검사 3) 기타 검사 (근전도, 내시경 등) 4) 수액 및 영양제 5) 일반 치료 약물

6) 항암제 7) 소독 물품 (드레싱키트, 알콜솜 등) 8) 무균 물품 (장갑, 가운, 초음파 젤 등)

9) 시술용 의료기기 (카테터 등) 10) 수술용 의료기기 및 장비 11) 기타 (주관식 서술)

Q18. (Q16.에서 1을 선택한 경우) 해당 행위로 인해 환자에게 위해가 발생할 것이라 판단하십니까?

1) 예 2) 아니오 3) 잘 모르겠다.

Q19. (위에서 1을 선택한 경우) 해당 행위가 지속되는 원인은 무엇이라고 생각하십니까?

1) 정부 지침 2) 병원 방침 혹은 상급자 지시 3) 관행적 이유 4) 인식 부족 5) 기타 (주관식 서술)

대한전공의협의회

**2019년 전국 전공의 설문조사**

| 문항 | 응답 |
| --- | --- |
| 전반적인 근무 환경에 대한 만족도를 점수로 표시해주세요. | \| 매우 불만족 \| \| \| 매우 만족 \| \| \| \| --- \| --- \| --- \| --- \| --- \| --- \| \| 1 \| 2 \| 3 \| \| 4 \| 5 \| |
| 전공의 복지와 관련해 담당부서(교육수련부 등)의 지원 및 역할에 만족하십니까? | \| 매우 불만족 \| \| \| 매우 만족 \| \| \| \| --- \| --- \| --- \| --- \| --- \| --- \| \| 1 \| 2 \| 3 \| \| 4 \| 5 \| |
| 최근 6개월간, 선생님께서는 최대 연속 수련시간인 36시간을 초과한적이 한 번 이상 있습니까? | \| 예 \| 아니오 \| \| --- \| --- \| \|  \|  \| |
| 최근 6개월간, 선생님의 1주일 평균 업무시간은 얼마입니까? | 시간 / 주 |
| 1년간 실제로 사용할 수 있는 휴가는 총 며칠입니까? | 일 |
| 최근 6개월간, 전날 업무를 마친 후 다음 정규시작 전까지 휴식시간은 평균 몇 시간 이었습니까? | 시간 / 일 |
| 지난 1주일 동안, 30분 이상의 점심 식사 시간을 가졌던 경우가 일주일에 평균 몇 번이었습니까? | 번 / 주 |
| 선생님께서는 당직 근무 중 환자에게 문제가 발생했을 때 전문의에게 즉시 보고 및 적절한 자문을 구할 수 있습니까? | \| 예 \| 아니오 \| \| --- \| --- \| \|  \|  \| |
| 선생님께서는 환자에게 술기를 행할 때, 전문의에게 적절한 지도 및 감독을 받을 수 있습니까? | \| 매우 아니다 \| \| \| 매우 그렇다 \| \| \| \| --- \| --- \| --- \| --- \| --- \| --- \| \| 1 \| 2 \| 3 \| \| 4 \| 5 \| |
| 전공의 수련 프로그램을 마치고 나면 전문의로서의 충분한 역량을 키울 수 있을 것이라고 생각하십니까? | \| 매우 아니다 \| \| \| 매우 그렇다 \| \| \| \| --- \| --- \| --- \| --- \| --- \| --- \| \| 1 \| 2 \| 3 \| \| 4 \| 5 \| |
| 선생님께서 수행하는 업무 중 수련과 관련 없는 업무가 차지하는 비중은 얼마입니까? | % |
| 선생님께서는 당직 근무 시 최대 몇 명의 환자를 담당합니까? | 명 |
| 선생님께서는 정규 근무 시 입원 환자를 평균 몇 명 담당합니까? | 명 |
| 선생님께서 지식 또는 술기를 배우고자 할 때 기회가 충분히 제공됩니까? | \| 매우 아니다 \| \| \| 매우 그렇다 \| \| \| \| --- \| --- \| --- \| --- \| --- \| --- \| \| 1 \| 2 \| 3 \| \| 4 \| 5 \| |
| 선생님은 소속된 병원의 수련환경에 만족하십니까? | \| 매우 불만족 \| \| \| 매우 만족 \| \| \| \| --- \| --- \| --- \| --- \| --- \| --- \| \| 1 \| 2 \| 3 \| \| 4 \| 5 \| |
| 선생님의 병원은 현재 교육수련 목표에 따른 수련과정을 제공하고 있습니까? | \| 매우 아니다 \| \| \| 매우 그렇다 \| \| \| \| --- \| --- \| --- \| --- \| --- \| --- \| \| 1 \| 2 \| 3 \| \| 4 \| 5 \| |

대한전공의협의회

**2018년 전국 전공의 설문조사**

| 문항 | 응답 |
| --- | --- |
| 귀하의 전반적인 근무환경에 만족하십니까? | \| 매우 불만족 \| \| \| 매우 만족 \| \| \| \| --- \| --- \| --- \| --- \| --- \| --- \| \| 1 \| 2 \| 3 \| \| 4 \| 5 \| |
| 귀하가 근무하는 병원 내 전반적인 근로 시설에 만족하십니까? | \| 매우 불만족 \| \| \| 매우 만족 \| \| \| \| --- \| --- \| --- \| --- \| --- \| --- \| \| 1 \| 2 \| 3 \| \| 4 \| 5 \| |
| 귀하는 지난 4주 동안 최대 연속 수련시간인 36시간을 초과한 적이 한 번 이상 있습니까? | \| 예 \| 아니오 \| \| --- \| --- \| \|  \|  \| |
| 최근 6개월 간, 평균 1주일에 몇 시간 근무하십니까? | 시간 / 주 |
| 1년간 부여받은 휴가는 총 며칠 입니까? (주말 및 공휴일 제외) | 일 |
| 1개월 근무 시 휴일(24시간 출근하지 않는 날, 휴가 제외)은 평균 며칠입니까? | 일 |
| 1주일에 야간 당직근무(응급의학과의 경우 야간 근로)를 평균 몇 회 하십니까? | 회 / 주 |
| 당직 근무 종료 후 정규시작 전까지 실질적인 휴식시간은 몇 시간 입니까? | 시간 |
| 근무 중 귀하의 하루 평균 수면시간은 몇 시간 입니까? | 시간 |
| 30분 이상의 점심 식사 시간을 가졌던 경우가 일주일에 평균 몇 번이었습니까? | 회 / 주 |
| 귀하께서 무면허진료보조인력 (UA, Unlicensed Assistant)로 인해 교육적 기회를 박탈당했다고 느낀 적이 있습니까? | \| 예 \| 아니오 \| \| --- \| --- \| \|  \|  \| |
| 귀하의 병원에 무면허진료보조인력 (UA, Unlicensed Assistant)가 있습니까? | \| 예 \| 아니오 \| \| --- \| --- \| \|  \|  \| |
| 지도전문의는 학습 과정에 효과적으로 기여하고 있습니까? | \| 매우 아니다 \| \| \| 매우 그렇다 \| \| \| \| --- \| --- \| --- \| --- \| --- \| --- \| \| 1 \| 2 \| 3 \| \| 4 \| 5 \| |
| 전공의 수련 프로그램을 성실히 마치고 나면 전문의로서 단독으로 일하는데 역량이 충분할 것이라고 생각하십니까? | \| 매우 아니다 \| \| \| 매우 그렇다 \| \| \| \| --- \| --- \| --- \| --- \| --- \| --- \| \| 1 \| 2 \| 3 \| \| 4 \| 5 \| |
| 전체 업무 중 수련과 관련 없는 업무가 차지하는 비중이 얼마나 됩니까? | % |
| 당직 근무 시 최대 몇 명의 환자를 담당합니까? | 명 |
| 정규 근무 시 주치의를 맡은 경우 입원 환자를 평균 몇 명 담당합니까? | 명 |
| 최근 1년 간 술기 교육을 받은 후, 적절한 빈도로 직접 수행해 볼 기회가 있었습니까? | \| 예 \| 아니오 \| 미해당 \| \| --- \| --- \| --- \| \|  \|  \|  \| |
| 최근 2달 간 경험한 전공의 수련 프로그램에 얼마나 만족하십니까? | \| 매우 불만족 \| \| \| 매우 만족 \| \| \| \| --- \| --- \| --- \| --- \| --- \| --- \| \| 1 \| 2 \| 3 \| \| 4 \| 5 \| |
| 전공의가 수행해야 할 업무를 상세히 기술한 문서(직무기술서)가 있습니까? | \| 예 \| 아니오 \| 모름 \| \| --- \| --- \| --- \| \|  \|  \|  \| |
| 전공의가 해당 업무를 통해 배우기로 되어 있는 지식, 기술, 태도를 갖추는데 필요한 학습 과정이 적절하게 구성되어 있습니까? | \| 매우 아니다 \| \| \| 매우 그렇다 \| \| \| \| --- \| --- \| --- \| --- \| --- \| --- \| \| 1 \| 2 \| 3 \| \| 4 \| 5 \| |

대한전공의협의회

**2017년 전국 전공의 설문조사**

| 문항 | 응답 |
| --- | --- |
| 전반적인 근무환경에 만족한다. | \| 매우 아니다 \| \| \| 매우 그렇다 \| \| \| \| --- \| --- \| --- \| --- \| --- \| --- \| \| 1 \| 2 \| 3 \| \| 4 \| 5 \| |
| 기타 전공의 복지 환경에 만족한다. | \| 매우 아니다 \| \| \| 매우 그렇다 \| \| \| \| --- \| --- \| --- \| --- \| --- \| --- \| \| 1 \| 2 \| 3 \| \| 4 \| 5 \| |
| 귀하는 지난 4주 동안 최대 연속 수련시간인 36시간을 초과한 적이 있습니까? | \| 예 \| 아니오 \| \| --- \| --- \| \|  \|  \| |
| 1주일에 평균 몇 시간 근무하십니까? | 시간 / 주 |
| 지난해(2016년) 귀하께서 전공의(인턴, 레지던트)로 근무하셨다면 실제 휴가를 며칠 가셨습니까? | 일 |
| 1개월 근무 시 평균 휴일(24시간 출근하지 않는 날, 휴가 제외)은 며칠이나 부여받습니까? | 일 / 달 |
| 야간이나 휴일에 당직근무(응급의학과의 경우 야간 근로)를 주 몇 회나 하십니까? | 회 / 주 |
| 당직 근무 종료 후 정규시작 전까지 실질적으로 휴게시간이 얼마나 보장되십니까? | 시간 |
| 1주일 간 귀하의 하루 평균 수면시간은 몇 시간 입니까? | 시간 / 일 |
| 점심 식사시간에 식사를 할 수 있는 경우가 일주일에 몇 번이나 되십니까? | 회 / 주 |
| 귀하께서 무면허진료보조인력 (UA, Unlicensed Assistant)로 인해 교육적 기회를 박탈당했다고 느낀 적이 있습니까? | \| 예 \| 아니오 \| \| --- \| --- \| \|  \|  \| |
| 귀하의 병원에 무면허진료보조인력 (UA, Unlicensed Assistant)가 있습니까? | \| 예 \| 아니오 \| \| --- \| --- \| \|  \|  \| |
| 귀하가 당직 근무 시 응급환자에 대해 담당교수와 논의하려고 할 때 신속하고 원활하게 소통했다고 생각하는 비율은 몇 % 입니까? | % |
| 지도전문의에게 적절한 교육적 지도를 받고 있다. | \| 매우 아니다 \| \| \| 매우 그렇다 \| \| \| \| --- \| --- \| --- \| --- \| --- \| --- \| \| 1 \| 2 \| 3 \| \| 4 \| 5 \| |
| 현재 전공의 수련이 수련 직후 봉직을 하거나 개원하는데 충분하다. | \| 매우 아니다 \| \| \| 매우 그렇다 \| \| \| \| --- \| --- \| --- \| --- \| --- \| --- \| \| 1 \| 2 \| 3 \| \| 4 \| 5 \| |
| 귀하의 ‘정규’ 업무 중 전공의 수련과 관련 없는 업무가 차지하는 비중이 얼마나 되십니까? | % |
| 당직 근무 시 주치의를 맡은 경우 환자를 평균 몇 명 담당합니까? | 명 |
| 정규 근무 시 주치의를 맡은 경우 입원 환자는 평균 몇 명 담당합니까? | 명 |
| 임상능력 함양을 위한 참여 기회(시술 참여, 의사결정과정 참여 등)가 있다. | \| 매우 아니다 \| \| \| 매우 그렇다 \| \| \| \| --- \| --- \| --- \| --- \| --- \| --- \| \| 1 \| 2 \| 3 \| \| 4 \| 5 \| |
| 전반적인 교육환경에 만족한다. | \| 매우 아니다 \| \| \| 매우 그렇다 \| \| \| \| --- \| --- \| --- \| --- \| --- \| --- \| \| 1 \| 2 \| 3 \| \| 4 \| 5 \| |
| 연차별(인턴 포함)로 수련 교육목표가 제시되어 있다. | \| 매우 아니다 \| \| \| 매우 그렇다 \| \| \| \| --- \| --- \| --- \| --- \| --- \| --- \| \| 1 \| 2 \| 3 \| \| 4 \| 5 \| |
| 연차별 수련이 체계적으로 진행되고 있다. | \| 매우 아니다 \| \| \| 매우 그렇다 \| \| \| \| --- \| --- \| --- \| --- \| --- \| --- \| \| 1 \| 2 \| 3 \| \| 4 \| 5 \| |

대한전공의협의회

**2016년 전국 전공의 설문조사**

| 문항 | 응답 |
| --- | --- |
| 전반적인 근무환경에 만족한다. | \| 매우 아니다 \| \| \| 매우 그렇다 \| \| \| \| --- \| --- \| --- \| --- \| --- \| --- \| \| 1 \| 2 \| 3 \| \| 4 \| 5 \| |
| 기타 전공의 복지 환경에 만족한다. | \| 매우 아니다 \| \| \| 매우 그렇다 \| \| \| \| --- \| --- \| --- \| --- \| --- \| --- \| \| 1 \| 2 \| 3 \| \| 4 \| 5 \| |
| 귀하는 지난 1주일간 최대 연속 수련시간이 36시간을 초과한 적이 있습니까? | \| 예 \| 아니오 \| \| --- \| --- \| \|  \|  \| |
| 1주일에 평균 몇 시간 근무하십니까? | 시간 / 주 |
| 지난해(2015년) 귀하께서 전공의(인턴, 레지던트)로 근무하셨다면 실제 휴가를 며칠 가셨습니까? (주말 및 공휴일 제외) | 일 |
| 1개월 근무 시 평균 휴일(off, 휴가 제외)은 며칠이나 부여받습니까? | 일 / 달 |
| 당직 근무(24시간 근무 or 야간근무)를 주 몇 회나 하십니까? | 회 / 주 |
| 당직 근무 종료 후 정규시작 전까지 실질적으로 휴게시간이 얼마나 보장되십니까? | 시간 |
| 지난 1주일 간 귀하의 하루 평균 수면시간은 몇 시간 이었습니까? | 시간 / 일 |
| 귀하께서 PA(Physician Assistant)로 인해 교육적 기회를 박탈당했다고 느낀 적이 있습니까? | \| 예 \| 아니오 \| \| --- \| --- \| \|  \|  \| |
| 귀하의 병원에 PA(Physician Assistant)가 있습니까? | \| 예 \| 아니오 \| \| --- \| --- \| \|  \|  \| |
| 귀하가 당직일 때 응급환자에 대한 논의가 필요한 경우 담당 교수와 신속하고 원활한 소통이 가능합니까? | \| 예 \| 아니오 \| \| --- \| --- \| \|  \|  \| |
| 지도전문의에게 적절한 교육적 지도를 받고 있다. | \| 매우 아니다 \| \| \| 매우 그렇다 \| \| \| \| --- \| --- \| --- \| --- \| --- \| --- \| \| 1 \| 2 \| 3 \| \| 4 \| 5 \| |
| 현재 전공의 수련이 수련 직후 봉직을 하거나 개원하는데 충분하다. | \| 매우 아니다 \| \| \| 매우 그렇다 \| \| \| \| --- \| --- \| --- \| --- \| --- \| --- \| \| 1 \| 2 \| 3 \| \| 4 \| 5 \| |
| 본인의 업무 중 전공의 수련과 관련 없는 업무가 차지하는 비중이 얼마나 되십니까? | % |
| 임상능력 함양을 위한 참여 기회(시술 참여, 의사결정과정 참여 등)가 있다. | \| 매우 아니다 \| \| \| 매우 그렇다 \| \| \| \| --- \| --- \| --- \| --- \| --- \| --- \| \| 1 \| 2 \| 3 \| \| 4 \| 5 \| |
| 전반적인 교육환경에 만족한다. | \| 매우 아니다 \| \| \| 매우 그렇다 \| \| \| \| --- \| --- \| --- \| --- \| --- \| --- \| \| 1 \| 2 \| 3 \| \| 4 \| 5 \| |
| 연차별(인턴 포함)로 수련 받아야 할 항목이 제시되어 있다. | \| 매우 아니다 \| \| \| 매우 그렇다 \| \| \| \| --- \| --- \| --- \| --- \| --- \| --- \| \| 1 \| 2 \| 3 \| \| 4 \| 5 \| |
| 제시된 연차별 수련교육과정(인턴 포함)이 체계적 진행되고 있다. | \| 매우 아니다 \| \| \| 매우 그렇다 \| \| \| \| --- \| --- \| --- \| --- \| --- \| --- \| \| 1 \| 2 \| 3 \| \| 4 \| 5 \| |
